# Opposing roles of DGAT‐mediated lipid droplet biogenesis in the regulation of ferroptosis sensitivity

**DOI:** 10.1111/febs.70467

**Published:** 2026-02-23

**Authors:** Ana Kump, Leja Perne, Špela Koren, Eva Jarc Jovičić, Nastja Feguš, Carina Pinto Kozmus, Michele Wölk, Kristyna Brejchova, Maria Fedorova, Ondrej Kuda, Toni Petan

**Affiliations:** ^1^ Department of Molecular and Biomedical Sciences Jožef Stefan Institute Ljubljana Slovenia; ^2^ Jožef Stefan International Postgraduate School Ljubljana Slovenia; ^3^ Center of Membrane Biochemistry and Lipid Research, Faculty of Medicine Carl Gustav Carus of TU Dresden Dresden Germany; ^4^ Laboratory of Metabolism of Bioactive Lipids, Institute of Physiology of the Czech Academy of Sciences Prague Czechia

**Keywords:** diacylglycerol acyltransferase, fatty acids, ferroptosis, lipid droplets, lipid peroxidation, lipidomics

## Abstract

Lipid droplets (LDs) are dynamic fat storage organelles involved in fatty acid metabolism, signaling, and trafficking. By storing polyunsaturated fatty acids (PUFAs) in the form of neutral lipids, LDs can either mitigate or exacerbate lipotoxic damage. However, their role in regulating cellular fatty acid distribution, membrane unsaturation, and ferroptosis susceptibility remains poorly understood. Here, we show that inhibition of diacylglycerol acyltransferase (DGAT)‐mediated LD biogenesis in PUFA‐supplemented triple‐negative breast cancer cells triggers widespread lipidome reorganization and membrane phospholipid acyl‐chain remodeling, promoting lipid peroxidation and ferroptosis sensitivity. Lipidomic analyses reveal that LDs efficiently sequester exogenous PUFAs within triacylglycerols and cholesteryl esters, significantly altering neutral lipid unsaturation profiles. When LD formation is impaired by DGAT inhibition, PUFAs are redistributed into membrane ester and ether glycerophospholipids, enhancing overall membrane unsaturation, lipid peroxidation, and increasing ferroptosis susceptibility, even in the absence of additional ferroptosis inducers. In contrast, in human lung adenocarcinoma cells, LDs exhibit a dual, context‐dependent role in ferroptosis regulation, whereby exogenous PUFA levels and the extent of ferroptosis protection determine whether DGAT inhibition promotes or protects against cell death. The pro‐ferroptotic function of LDs predominates in these cells and is strongly enhanced by ferroptosis suppressor protein 1 (FSP1) deficiency, which amplifies lipid peroxidation within LDs and promotes its propagation to other cellular compartments. This study highlights LDs as multifaceted regulators of ferroptosis, interlinking metabolic and redox quality control mechanisms.

## Graphical abstract

Ferroptosis is driven by unrestricted peroxidation of polyunsaturated fatty acyl (PUFA) chains in membrane phospholipids. In triple‐negative breast cancer cells, lipid droplets (LDs) sequester dietary PUFAs, limiting their incorporation into membrane phospholipids, preventing mitochondrial damage, and reducing lipid peroxidation and ferroptosis. In contrast, lung adenocarcinoma cells lose this protective advantage because their LD‐stored lipids are readily oxidized, converting LDs into pro‐ferroptotic organelles that amplify lipid peroxidation and promote ferroptosis.

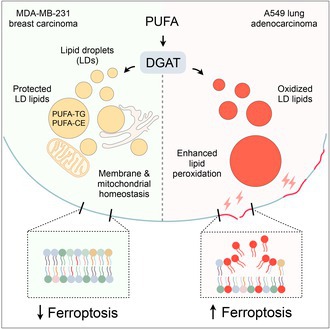

**Abbreviations**
7‐AAD: 7‐aminoactinomycin D
AA: arachidonic acid
ACSL: acyl‐CoA synthetase
AdA: adrenic acid
AGPAT: acylglycerol‐3‐phosphate acyltransferase
CAR: acylcarnitine
CE: cholesteryl ester
CoQ: coenzyme Q (ubiquinone)
CoQH_2_
: reduced coenzyme Q (ubiquinol)
DGAT: diacylglycerol acyltransferase
DHA: docosahexaenoic acid
DRP1: dynamin‐related protein 1
ER: endoplasmic reticulum
FA: fatty acid
FBS: fetal bovine serum
FSP1: ferroptosis suppressor protein 1
GPAT: glycerol‐3‐phosphate acyltransferase
GPX4: glutathione peroxidase 4
KEAP1: Kelch‐like ECH‐associated protein 1
LD: lipid droplet
LORA: lipid over‐representation analysis
LPCAT: lysophospholipid acyltransferase
MUFA: monounsaturated fatty acid
NRF2: nuclear factor erythroid 2‐related factor
OA: oleic acid
PC: phosphatidylcholine
PCA: principal component analysis
PE: phosphatidylethanolamine
PI: phosphatidylinositol
PL: glycerophospholipid
PS: phosphatidylserine
PUFA: polyunsaturated fatty acid
PUFA_2_‐PL: phospholipid with two PUFA chains
ROS: reactive oxygen species
RSL3: Ras‐selective lethal 3
SFA: saturated fatty acid
siRNA: small interfering RNA
TG: triacylglycerol
TMRM: tetramethylrhodamine methyl ester
TNBC: triple‐negative breast cancer

## Introduction

Fatty acids (FAs) are essential for cellular structure and metabolism. Within cells, FAs are rarely found in their free form; instead, they are typically incorporated into complex lipids such as triacylglycerols (TGs), glycerophospholipids (PLs), and sterols. Their presence in membrane glycerophospholipids is crucial for the biophysical properties and dynamics of cellular membranes as well as the function of membrane proteins [1]. FAs also serve as energy sources, transcriptional regulators, signaling molecules, and anchors for peripheral membrane proteins [2, 3, 4, 5]. When cells fail to sufficiently metabolize or store excess FAs, they accumulate and cause lipotoxicity—disrupting cellular homeostasis, inducing oxidative stress, impairing organelle function, and potentially triggering cell death [6]. FAs are primarily stored in their esterified forms as TGs and steryl esters within lipid droplets (LDs), dynamic organelles originating from the endoplasmic reticulum (ER) [7, 8]. LDs undergo continuous cycles of biogenesis and breakdown, balancing FA storage and release in response to current cellular needs [9, 10]. By sequestering excess FAs, LDs protect other organelles from lipotoxic damage, thus maintaining cellular homeostasis across various biological contexts [11, 12, 13, 14, 15]. Conversely, the regulated release of FAs from neutral lipids stored in LDs is crucial for fundamental processes such as energy production, membrane biogenesis, and redox homeostasis [15, 16]. However, the precise role of LDs in directing specific FAs to distinct cellular destinations for purposes like membrane lipid remodeling remains poorly understood.

The balance between saturated, unsaturated and polyunsaturated FAs (PUFAs) esterified in membrane PLs is crucial for proper membrane and organelle function [1]. PUFAs, such as arachidonic acid (AA; C20:4; ω‐6), adrenic acid (AdA; C22:4; ω‐6), and docosahexaenoic acid (DHA; C22:6; ω‐3), are key membrane components shaping its essential properties, such as bending, fluidity and thickness [17]. PUFAs are also substrates for controlled enzymatic oxygenation reactions, producing potent signaling molecules, such as eicosanoids, derived from PUFAs with 20 carbon atoms [18]. However, due to the weak bis‐allylic methylene groups in their structure, PUFAs are also highly susceptible to peroxidation, which can occur through non‐enzymatic, free radical‐mediated reactions (autoxidation), as well as through direct and indirect enzymatic mechanisms, mediated by lipoxygenases, cyclooxygenases, and cytochrome P450 enzymes [19, 20, 21, 22]. Unrestrained buildup of peroxidized PLs within cellular membranes can result in ferroptosis—a form of iron‐dependent, non‐apoptotic regulated cell death [19, 20, 23, 24, 25, 26, 27]. Ferroptosis essentially occurs when the accumulation of membrane lipid peroxides exceeds the capacity of cellular lipid quality control mechanisms, which continuously act to prevent the propagation of lipid peroxidation and to eliminate damaged lipids [22]. The primary cellular defense mechanism relies on glutathione peroxidase 4 (GPX4), which directly reduces toxic lipid hydroperoxides to harmless alcohols [28]. The action of GPX4 is complemented by ferroptosis suppressor protein 1 (FSP1), which regenerates the membrane‐resident radical‐trapping antioxidants ubiquinol (CoQH_2_) and vitamin K [29, 30, 31]. Ferroptosis is also regulated by lipid metabolism and remodeling enzymes, which dynamically control membrane lipid composition and PUFA content [32, 33, 34, 35, 36, 37, 38]. Accordingly, supplementing cancer cells with PUFAs can result in ferroptosis and dietary PUFA interventions compromise tumor growth by promoting ferroptosis [39, 40, 41, 42, 43, 44].

The accumulation of PUFA‐containing TGs within LDs can reduce lipotoxic damage by creating a protective neutral lipid environment that potentially limits PUFA peroxidation [45, 46]. This sequestration can also decrease PUFA incorporation into membrane lipids, thereby reducing lipid peroxidation and ferroptosis susceptibility [42, 45, 46, 47, 48, 49]. Conversely, the breakdown of PUFA‐loaded LDs can also promote oxidative stress and ferroptotic cell death by delivering PUFAs to membrane phospholipids and other organelles [37, 47, 50, 51, 52]. PUFA‐rich neutral lipids within LDs can themselves undergo oxidation, which is counteracted by LD‐localized FSP1 [53]. In FSP1‐deficient cells, these oxidized lipids can act as a source of lethal lipid peroxidation, leading to plasma membrane rupture and ferroptotic death [53]. Thus, the role of LDs in modulating PUFA‐induced cytotoxicity and ferroptosis depends on multiple factors, including cell type‐specific metabolic and redox programs, as well as environmental conditions that define lipid availability and cellular state.

We have previously shown that PUFA supplementation in triple‐negative breast cancer (TNBC) cells exerts a dual effect, promoting either cell survival and proliferation or oxidative stress and death, depending on concentration [47, 54]. Here, we investigated how DGAT‐mediated TG synthesis and LD biogenesis shape lipidome remodeling, peroxidation, and ferroptotic cell death in PUFA‐supplemented TNBC cells. We found that inhibiting LD biogenesis triggered extensive lipidome reorganization and membrane acyl‐chain remodeling, enhancing lipid peroxidation and ferroptosis sensitivity even in the absence of canonical inducers. This ferroptosis‐prone state was characterized by enrichment of multiple PL classes with PUFAs. In contrast, in ferroptosis‐resistant lung adenocarcinoma cells, LDs played a context‐dependent role: depending on the mode of ferroptosis induction and the magnitude of PUFA‐induced cell damage, DGAT inhibition either sensitized cells to or protected them from ferroptosis.

## Results

### DGAT inhibition potentiates PUFA‐induced lipid peroxidation and ferroptosis

Our previous work demonstrated that PUFA supplementation induces LD accumulation in MDA‐MB‐231 TNBC cells, alongside effects ranging from protection against serum starvation to oxidative stress and cell death [47]. Here, we hypothesized that PUFA supplementation promotes membrane lipid peroxidation and ferroptosis and that impairing LD biogenesis exacerbates these effects. To test this, we supplemented MDA‐MB‐231 cells with DHA and assessed the impact of DGAT inhibition on LD accumulation, oxidative stress, lipid reactive oxygen species (ROS) generation and cell viability.

**Fig. 1:**
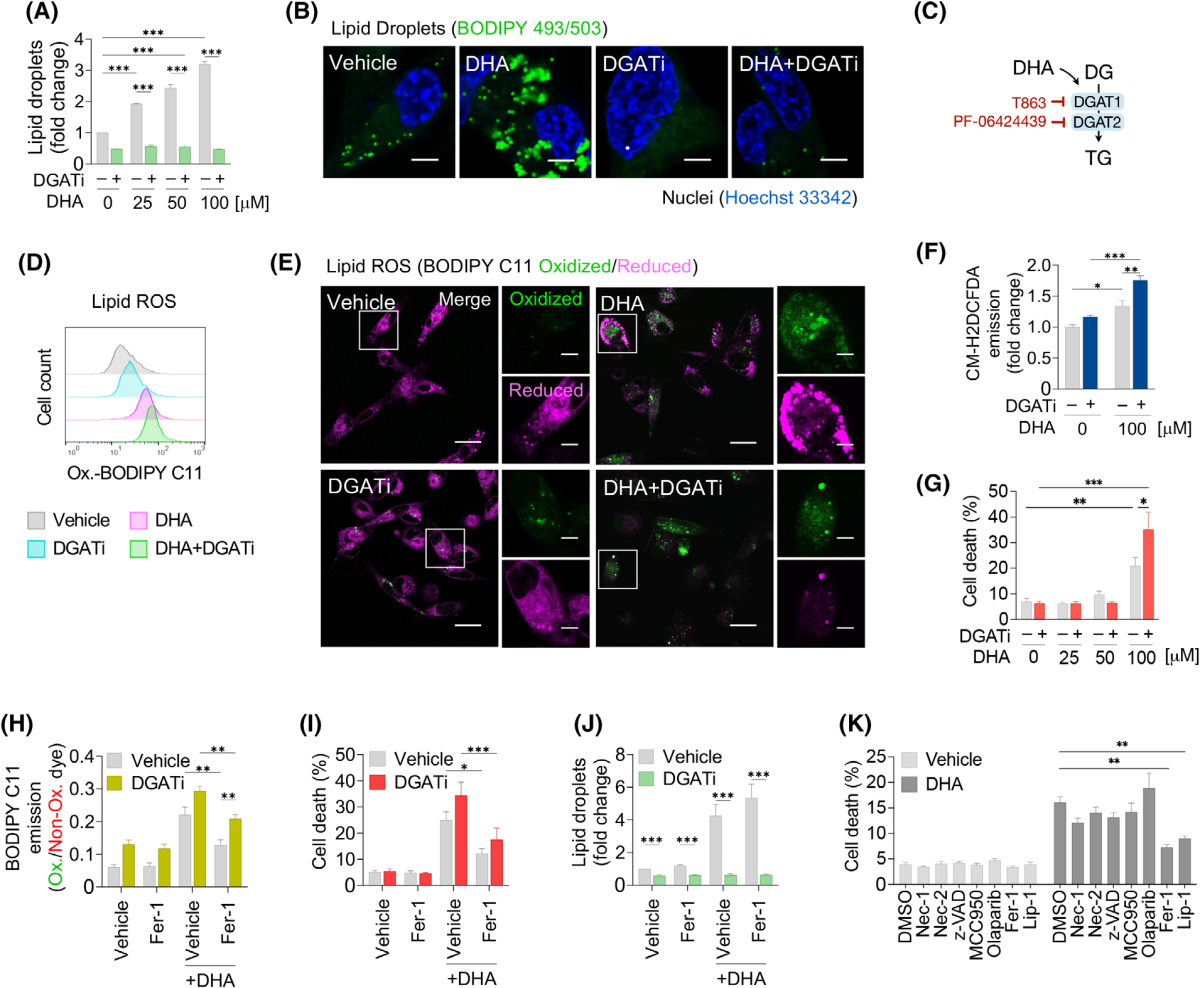
DGAT inhibition potentiates PUFA‐induced lipid peroxidation and ferroptosis. (A) Lipid droplet levels measured by Nile Red staining in MDA‐MB‐231 cells treated with 25–100 μm docosahexaenoic acid (DHA) for 24 h, with or without 20 μm DGAT1 and 20 μm DGAT2 inhibitors (DGATi). (B) Representative confocal microscopy images of MDA‐MB‐231 cells treated with 100 μm DHA for 24 h, with or without DGATi. Lipid droplets were stained with BODIPY 493/503 (green) and nuclei with Hoechst 33342 (blue). Scale bar, 5 μm. (C) General scheme of the last step in TG synthesis mediated by DGAT1 and DGAT2 enzymes, inhibited by specific inhibitors T863 and PF‐06424439, respectively. (D) Lipid peroxidation assessed by BODIPY C11 581/591 probe using flow cytometry in MDA‐MB‐231 cells following the treatment described in (B). The *x*‐axis on the histogram represents fluorescence intensity of oxidized BODIPY C11 581/591 and the *y*‐axis shows cell count. (E) Representative confocal microscopy images of MDA‐MB‐231 cells showing oxidized (green) and reduced (magenta) fluorescent signals of the BODIPY C11 581/591 probe induced by the treatment described in (B). Boxed insets show an area approximately covering a single cell displayed in separate fluorescent channels. Scale bars, 20 μm and 5 μm (inset). (F) Total reactive oxygen species (ROS) measured by CM‐H2DCFDA in MDA‐MB‐231 cells following the treatment described in (B). (G) Cell death (%) determined by TMRM/YO‐PRO‐1 staining in MDA‐MB‐231 cells following the treatment described in (A). (H) Lipid peroxidation measured by BODIPY C11 581/591 in MDA‐MB‐231 cells treated with 100 μm DHA and/or 5 μm ferrostatin‐1 (Fer‐1) for 24 h, with or without DGATi. (I) Cell death (%) determined by TMRM/YO‐PRO‐1 staining in MDA‐MB‐231 cells following the treatment described in (H). (J) Lipid droplet levels measured by Nile Red staining in MDA‐MB‐231 cells following the treatment described in (H). (K) Cell death (%) determined by TMRM/YO‐PRO‐1 staining in MDA‐MB‐231 cells following treatment with 100 μm DHA for 24 h, with or without 10 μm necrostatin‐1 (Nec‐1), 5 μm necrostatin‐2 (Nec‐2), 50 μm z‐VAD‐fmk (z‐VAD), 5 μm MCC950, 2.5 μm olaparib, 5 μm Fer‐1, 5 μm liproxstatin‐1 (Lip‐1). Data are presented as mean ± SEM of three (A, K), four (F), five (G, J), and six (H, I) independent biological replicates. Microscopic images (B, E) are representative of three independent biological replicates. Significance was calculated by two‐way ANOVA with Tukey adjustment (A, F–J) or unpaired *t*‐test (K). *P* > 0.05 = ns; *P < 0.05; **P < 0.01; ***P < 0.001.

DHA supplementation induced a concentration‐dependent 2‐ to 3.5‐fold increase in neutral lipid content (Fig. 1A), which corresponded with substantial LD accumulation observed by confocal microscopy (Fig. 1B). To determine if DHA promotes LD biogenesis through DGAT enzymes, we used inhibitors targeting DGAT1 (T863) and DGAT2 (PF‐06424439) (Fig. 1C). DGAT inhibition significantly reduced neutral lipid levels and fully abrogated DHA‐induced LD accumulation (Fig. 1A,B). Next, we evaluated oxidative stress and lipid peroxidation using CM‐H2DCFDA as a general ROS sensor and BODIPY 581/591 C11 as a lipid ROS sensor. DHA supplementation triggered a concentration‐dependent rise in both lipid ROS and total ROS levels (Fig. 1D–F; Fig. S1A), effects that were further amplified by DGAT inhibition. Importantly, DGAT inhibition potentiated DHA‐induced cell death (Fig. 1G), indicating that LD biogenesis protects against PUFA‐induced oxidative damage and cell death.

To confirm that ferroptosis underlies DHA‐induced cytotoxicity, we treated cells with ferrostatin‐1 and liproxstatin‐1, lipophilic radical‐trapping agents and specific ferroptosis inhibitors [55, 56, 57]. Ferrostatin‐1 reduced DHA‐induced lipid ROS accumulation (Fig. 1H) and cell death (Fig. 1I), without affecting LD levels (Fig. 1J). Liproxstatin‐1 had a similar potency as ferrostatin‐1 in reducing cell death upon DHA supplementation, whereas inhibitors of other cell death pathways, including necroptosis (necrostatin‐1, necrostatin‐2), apoptosis (z‐VAD‐fmk), pyroptosis (MCC950), and parthanatos (olaparib), failed to rescue cell viability upon DHA treatment (Fig. 1K), confirming ferroptosis as the dominant mode of death. Moreover, ferrostatin‐1 attenuated the increase in lipid peroxidation (Fig. 1H) and cell death (Fig. 1I) caused by DGAT inhibition, further implicating LD biogenesis in modulation of ferroptosis susceptibility. To corroborate that the observed effects of DHA depend on its polyunsaturation, we used oleic acid (OA; C18:1, ω‐9), a monounsaturated fatty acid (MUFA) known to resist oxidation and enhance ferroptosis resistance when incorporated into membrane phospholipids [33]. Supplementing cells with OA promoted LD accumulation (Fig. S1B) but did not induce cell death, even with DGAT inhibition (Fig. S1C). Together, these results demonstrate that DGAT‐mediated LD biogenesis buffers excess PUFAs and mitigates lipid peroxidation, thereby protecting MDA‐MB‐231 cells from ferroptotic death.

### DHA supplementation remodels LD‐stored neutral lipids

**Fig. 2:**
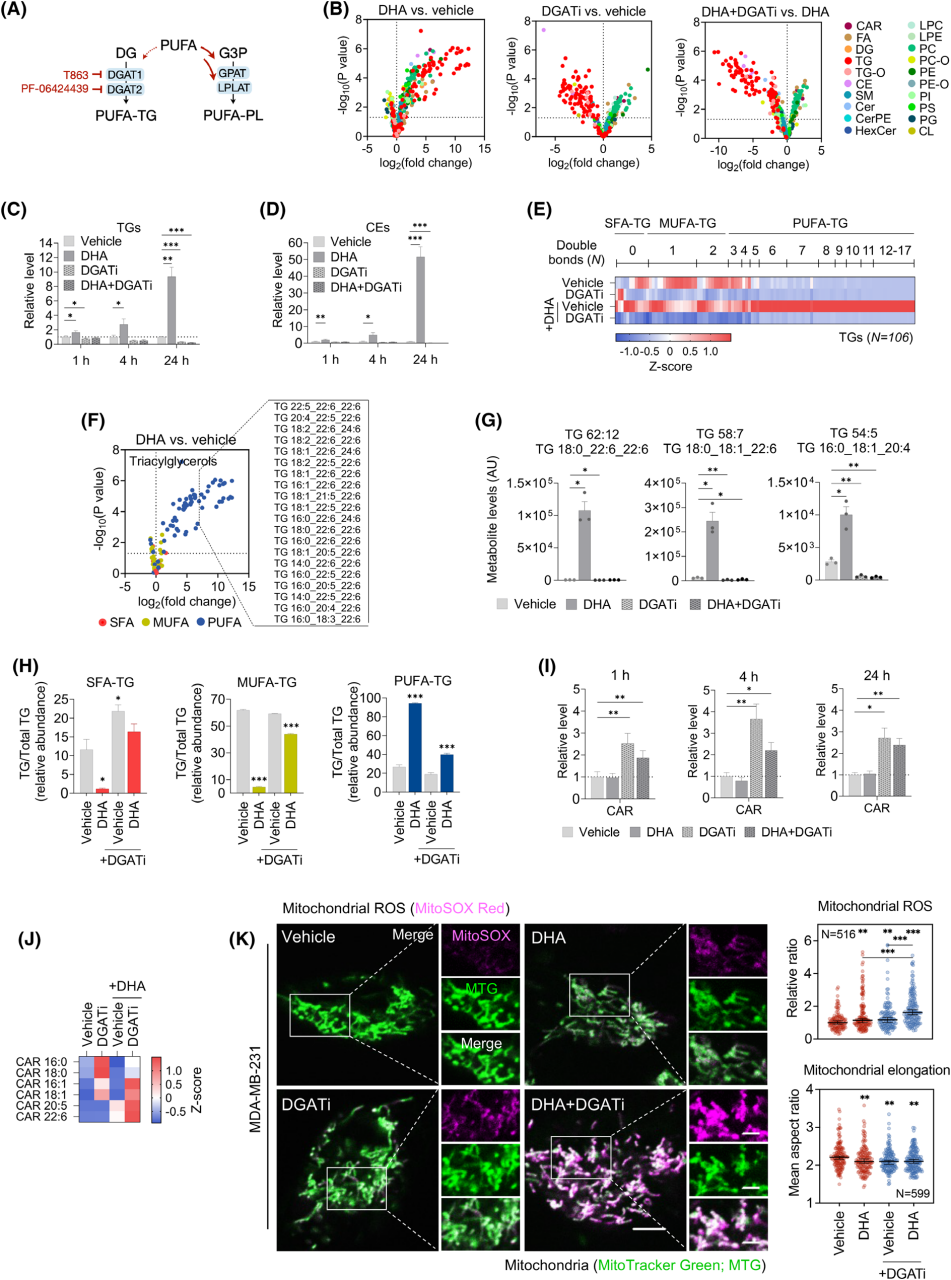
DHA supplementation remodels LD‐stored neutral lipids. (A) Model illustrating the proposed mechanism of DGAT‐mediated sensitization to PUFA‐induced ferroptosis. (B) Volcano plots showing lipids altered by 24 h treatment of MDA‐MB‐231 cells with 25 μm DHA, 20 μm DGAT1, and 20 μm DGAT2 inhibitors (DGATi) and a combination of both treatments. Lipids are colored by lipid class as indicated in the legend. (C, D) Relative total levels of TGs (C) and CEs (D) in MDA‐MB‐231 cells treated with 25 μm DHA for 1, 4, and 24 h, with or without DGATi. (E) Heatmap showing relative abundance of TGs in MDA‐MB‐231 cells following the treatment described in (B), sorted by degree of unsaturation (number of double bonds) and categorized as SFA‐TG (containing only saturated FAs), MUFA‐TGs (containing saturated and monounsaturated FAs), or PUFA‐TG (containing polyunsaturated FAs with at least 2 double bonds per chain). (F) Volcano plot of TG species altered by 24 h treatment with 25 μm DHA in MDA‐MB‐231 cells. TGs are categorized and colored by unsaturation as indicated in the legend. The 20 most upregulated TGs are highlighted and labeled. (G) Abundance of selected PUFA‐TGs in MDA‐MB‐231 cells following the treatment described in (B). Each point represents a biological replicate. (H) Relative abundance of SFA‐, MUFA‐, or PUFA‐containing TGs in MDA‐MB‐231 cells following the treatment described in (B). (I) Relative total levels of CAR in MDA‐MB‐231 cells following the treatment described in (C). (J) Heatmap showing the relative abundance of CARs in MDA‐MB‐231 cells after the same 24 h treatment as described in (B). (K) Representative confocal microscopy images of MDA‐MB‐231 cells stained with MitoSOX Red (mitochondrial ROS probe; magenta) and MitoTracker Green (mitochondria marker; green) following 24 h treatment with 50 μm DHA, with or without DGATi. Boxed insets show representative regions of a single cell displayed in separate and merged fluorescent channels. Scale bars, 5 μm and 2 μm (inset). Quantification of mitochondrial ROS (MitoSOX/MitoTracker ratio) (*n* = 516) and mitochondrial elongation (mean aspect ratio) (*n* = 599) is shown. Each point represents an individual cell. Data are presented as mean ± SEM (C, D, G–I) or median ± 95% CI (K) from two (K) or three (C, D, G–I) independent biological replicates. Microscopic images (K) are representative of two independent biological replicates. Data for volcano plots (B, F) were generated using log_2_‐transformed fold‐change values and multiple unpaired *t*‐tests (*P* < 0.05). Data for heat maps (E, J) were *z*‐score normalized. Significance was calculated by unpaired *t*‐test. *P* > 0.05 = ns; *P < 0.05; **P < 0.01; ***P < 0.001. CAR, acylcarnitine; CE, cholesterol ester; Cer, ceramide; CerPE, ceramide phosphoethanolamine; CL, cardiolipin; DG, diacylglycerol; FA, fatty acid; G3P, glycerol‐3‐phosphate; GPAT, glycerol‐3‐phosphate acyltransferase; HexCer, hexosylceramide; LPC, lyso‐phosphatidylcholine; LPE, lyso‐phosphatidylethanolamine; LPLAT, lysophospholipid acyltransferase; PAP, phosphatidic acid phosphatase; PC, phosphatidylcholine; PC‐O, ether‐linked phosphatidylcholine; PE, phosphatidylethanolamine; PE‐O, ether‐linked phosphatidylethanolamine; PG, phosphatidylglycerol; PI, phosphatidylinositol; PS, phosphatidylserine; SM, sphingomyelin; TG, triacylglycerol; TG‐O, ether‐linked triacylglycerol.

Our data show that DGAT inhibition, and the resulting impairment of LD biogenesis, sensitizes MDA‐MB‐231 TNBC cells to DHA‐induced ferroptosis. A likely explanation is that blocking TG synthesis prevents sequestration of exogenous DHA into neutral lipids, enhancing its incorporation into membrane PLs and promoting lipid peroxidation (Fig. 2A). To test this, we analyzed lipidome remodeling under low, sublethal DHA exposure (25 μM) to minimize lipotoxicity [47] (Fig. 1G). Cells were treated with DHA for 1, 4, and 24 h, with or without DGAT inhibitors, followed by LC–MS/MS‐based lipidomics.

Global lipidomic analysis across 565 species revealed substantial remodeling under all conditions, with 167, 267, and 302 significantly altered lipids in response to DGAT inhibition, DHA supplementation, or combined treatment, respectively (Fig. 2B). In DHA‐supplemented cells, neutral lipids (TGs, CEs) were elevated, which was fully suppressed by DGAT inhibition (Fig. 2B). Time‐course analysis confirmed progressive, DGAT‐dependent increase in total TG and CE levels with DHA supplementation (Fig. 2C,D). Focusing on TG acyl‐chain remodeling, DHA supplementation preferentially increased TGs with five or more double bonds (Fig. 2E), including TG species containing one, two, or three PUFA chains (Fig. 2F,G). This confirmed efficient DHA incorporation into TGs, though other non‐DHA‐containing PUFA‐TGs (e.g., arachidonic acid (AA)‐containing), as well as several non‐PUFA TGs, were also elevated (Fig. 2F,G; Fig. S2A). DGAT inhibition reduced the abundance of nearly all TG species, abolishing the DHA‐induced accumulation (Fig. 2E,G; Fig. S2A–C). Similarly, DHA‐induced PUFA‐CE enrichment was completely blocked by DGAT inhibition (Fig. S2D,E). To assess global changes in TG unsaturation, we grouped TGs by acyl‐chain unsaturation. DHA supplementation shifted TG composition from 26 to 94% PUFA‐TGs (Fig. 2H; Fig. S2F), accompanied by declines in SFA‐TGs (10 to 4%) and MUFA‐TGs (60 to 6%), highlighting the remarkable plasticity of LDs in storing excess PUFAs. DGAT inhibition almost completely prevented this shift, blocking DHA incorporation into TGs and restoring the fatty acid composition of residual TGs to near control levels.

Beyond neutral lipids, DHA supplementation modestly elevated diacylglycerols, sphingomyelins, and unesterified DHA (Fig. S2G–I). DGAT inhibition further increased free DHA and diacylglycerols, suggesting persistent DHA excess and ongoing lipid remodeling (Fig. S2G,I). Strikingly, only DGAT inhibition induced a strong accumulation of acylcarnitines (Fig. 2I,J), particularly PUFA‐containing acylcarnitines in DHA‐supplemented cells (Fig. 2J; Fig. S2J). This buildup suggests mitochondrial FA import overload and dysfunction [58, 59]. Consistent with this, DGAT inhibition increased mitochondrial ROS, which was further exacerbated by DHA supplementation (Fig. 2K). Mitochondrial fragmentation, another hallmark of mitochondrial dysfunction recently implicated in ferroptosis [60, 61], was also evident in DHA‐supplemented, DGAT inhibitor‐treated cells (Fig. 2K). Altogether, these results indicate that blocking TG synthesis redirects excess DHA toward alternative pathways, including diacylglycerol and acylcarnitine production, driving elevated glycerophospholipid remodeling, mitochondrial stress and increased susceptibility to ferroptosis.

### DGAT inhibition induces broad lipidome remodeling and enriches membrane phospholipids with PUFAs

**Fig. 3:**
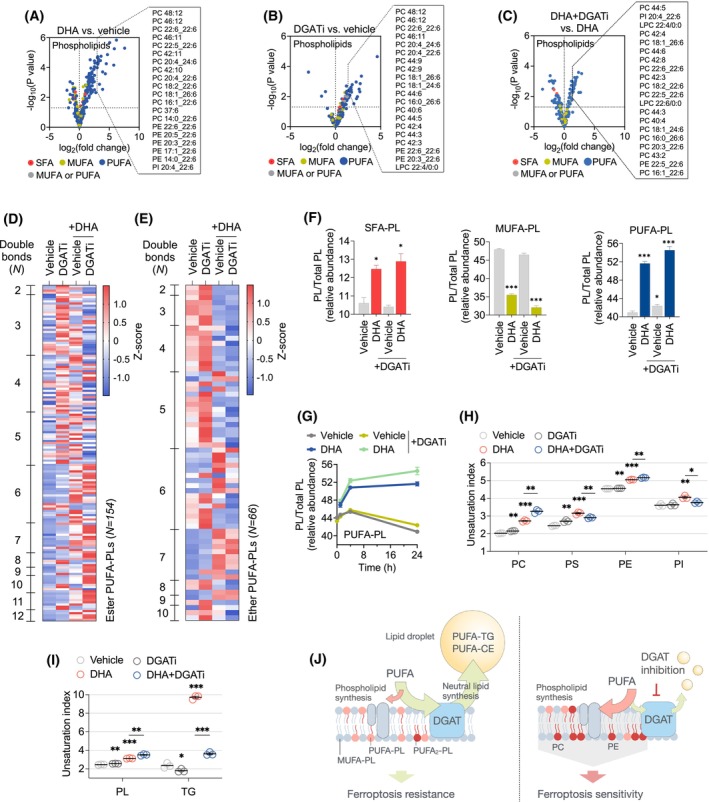
Inhibition of LD biogenesis leads to lipidome remodeling favoring PUFA enrichment in phospholipids. (A–C) Volcano plot of glycerophospholipids (PLs) altered by 24 h treatment of MDA‐MB‐231 cells with 25 μm docosahexaenoic acid (DHA), with or without 20 μm DGAT1 and 20 μm DGAT2 inhibitors (DGATi). PLs were categorized and colored as SFA (containing 0 double bonds in total), MUFA (containing 1 double bond in total), PUFA (containing > 2 double bonds in total), MUFA or PUFA (2 double bonds in total) as indicated in the legend. Data from three independent biological replicates were prepared using log_2_‐transformed fold‐change values and multiple unpaired *t*‐tests (*P* < 0.05). (D) Heatmap showing relative abundances of all quantified ester‐linked PLs that contain polyunsaturated (≥ 2 double bonds) fatty acyl chains (PUFA‐PLs) following the treatment described in (A). Lipids are sorted by degree of unsaturation (number of double bonds). Data were *z*‐score normalized. (E) Heatmap showing relative abundances of all quantified ether‐linked PLs that contain polyunsaturated (≥ 2 double bonds) fatty acyl chains (PUFA‐PLs) following the treatment described in (A). Lipids are sorted by degree of unsaturation (number of double bonds). Data were *Z*‐score normalized. (F) Relative abundance of SFA‐, MUFA‐, and PUFA‐containing PLs following the treatment described in (A). (G) Relative abundance of PUFA‐containing PLs following 1, 4, and 24 h treatment described in (A). (H) Abundance‐weighed unsaturation index of the major PL classes following the treatment described in (A). Each point represents a biological replicate. (I) Abundance‐weighed unsaturation index of the PL and TG lipid classes following the treatment described in (A). Each point represents a biological replicate. (J) Model summarizing the effects of DGAT inhibition on lipid remodeling underlying ferroptosis sensitization in PUFA‐supplemented cells. Data are presented as mean ± SEM (F–I) from three independent biological replicates. Significance was calculated by unpaired *t*‐test. *P* > 0.05 = ns; *P < 0.05; **P < 0.01; ***P < 0.001. PC, phosphatidylcholine; PE, phosphatidylethanolamine; PI, phosphatidylinositol; PS, phosphatidylserine; PUFA_2_‐PL, phospholipid with two PUFA chains.

To investigate whether PUFA incorporation into membrane PLs drives lipid peroxidation and ferroptosis sensitivity upon DGAT inhibition, we analyzed PL acyl‐chain remodeling following DHA supplementation and DGAT inhibition. Grouping PLs by unsaturation and using volcano plots we found that both DHA treatment and DGAT inhibition increased many PUFA‐PLs with various headgroups, each carrying one or two polyunsaturated acyl chains (Fig. 3A,B). Combining DHA supplementation with DGAT inhibition led to even greater PUFA‐PL enrichment (Fig. 3C). Heatmap analysis after z‐score normalization revealed distinct patterns: DGAT inhibition primarily upregulated PUFA‐PLs with 2–5 double bonds, including both ester‐ (Fig. 3D) and ether‐linked species (Fig. 3E). DHA supplementation alone increased PLs with 5–7 double bonds, while DHA combined with DGAT inhibition markedly increased a broader set of highly unsaturated PLs with 6–12 double bonds, particularly in ester‐linked species (Fig. 3D,E). Tracking the relative abundances of PLs grouped by their unsaturation revealed that PUFA‐PL levels increased over time with DHA supplementation, and DGAT inhibition further raised PUFA‐PLs in both control and in DHA‐supplemented cells (Fig. 3F,G; Fig. S3A). Interestingly, DHA supplementation also raised PLs containing only saturated fatty acyl (SFA) chains (SFA‐PLs). Together, these results show that DGAT inhibition and DHA supplementation synergistically remodel the membrane lipidome toward PUFA enrichment.

While PUFA‐phosphatidylethanolamine (PUFA‐PE) species were initially identified as key ferroptosis drivers [19, 62, 63], other PL classes also contribute to ferroptosis [21, 33, 35, 43, 64, 65, 66, 67]. To assess PUFA enrichment across different headgroups, we measured changes in the overall unsaturation within each PL class [68]. This analysis showed that DHA supplementation increased PUFA content across all PL classes, with the strongest enrichment observed in phosphatidylcholine (PC) and phosphatidylserine (PS) (Fig. 3H). DGAT inhibition alone increased PC, PS, and, to a lesser extent, PE unsaturation. Notably, in DHA‐treated cells, DGAT inhibition further increased PC and PE unsaturation but blunted PS and phosphatidylinositol (PI) remodeling, still driving a net rise in overall PL unsaturation (Fig. 3I). Lipid over‐representation analysis (LORA) [69] confirmed specific enrichment of long‐chain, highly unsaturated PC and PE species in DHA‐ and DGATi‐treated cells (Fig. S3B). At the individual lipid level, multiple PUFA‐PLs from all four PL classes, either containing one (PUFA_1_‐PL) or two (PUFA_2_‐PL) PUFA chains, including PE 18:0_22:6, PE 20:4_22:6, PC 22:6_22:6, and PI 20:4_22:6, were increased by DHA and further augmented by DGAT inhibition (Fig. S3C–E). Together, these findings show that ferroptosis sensitivity arises from broad remodeling across multiple PL classes, involving various PUFA‐PLs with one or two PUFA chains. Overall, our lipidomics reveal that DHA supplementation drives extensive PL remodeling and unsaturation and that DGAT inhibition further augments PUFA incorporation across different headgroups, promoting a ferroptosis‐prone lipidome (Fig. 3J).

### DGAT inhibition fails to promote DHA‐induced cytotoxicity in ferroptosis‐resistant cells

**Fig. 4:**
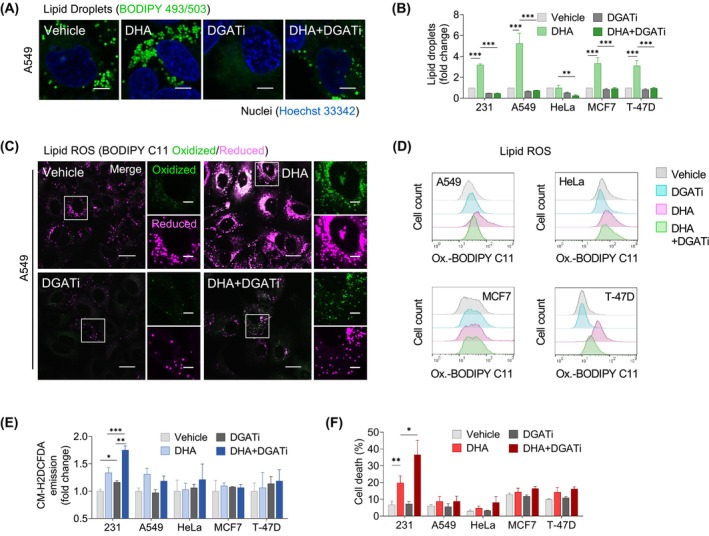
DGAT inhibition does not affect the viability of DHA‐supplemented ferroptosis‐resistant cells. (A) Representative confocal microscopy images of A549 cells treated with 100 μm docosahexaenoic acid (DHA) for 24 h, with or without 20 μm DGAT1 and 20 μm DGAT2 inhibitors (DGATi). Lipid droplets were stained with BODIPY 493/503 (green) and nuclei with Hoechst 33342 (blue). Scale bar, 5 μm. (B) Lipid droplet levels measured by Nile Red in indicated cell lines following the treatment described in (A). (C) Representative confocal microscopy images of A549 cells showing oxidized (green) and reduced (magenta) fluorescent signals of the BODIPY C11 581/591 probe induced by the treatment described in (A). Boxed insets show an area approximately covering a single cell displayed in separate fluorescent channels. Scale bars, 20 μm and 5 μm (inset). (D) Lipid peroxidation assessed by BODIPY C11 581/591 in indicated cell lines induced by the treatment described in (A). (E) Total reactive oxygen species (ROS) measured by CM‐H2DCFDA in indicated cell lines following the treatment described in (A). (F) Cell death (%) determined by TMRM/YO‐PRO‐1 staining in indicated cell lines following the treatment described in (A). Microscopic images are representative of three (A) or four (C) independent biological replicates. All other data are presented as mean ± SEM of three independent biological replicates. Significance was calculated by two‐way ANOVA with Tukey adjustment. *P* > 0.05 = ns; *P < 0.05; **P < 0.01; ***P < 0.001.

To assess whether DGAT inhibition sensitizes ferroptosis‐resistant cancer cells to DHA‐induced cell death, in addition to its effects in the ferroptosis‐sensitive MDA‐MB‐231 TNBC line, we selected four additional cancer cell lines with different origins, oncogenic drivers, metabolic profiles, and antioxidant capacities (Table S1) [29, 36, 37, 54, 63, 70, 71, 72, 73, 74, 75, 76, 77, 78, 79, 80, 81, 82, 83, 84, 85, 86, 87, 88, 89, 90, 91, 92, 93, 94]. The selected cell lines displayed distinct responses in LD accumulation following DHA supplementation. Lung adenocarcinoma A549 cells showed the most pronounced LD accumulation, with up to a 5‐fold increase in neutral lipid levels (Fig. 4A,B; Fig. S4A). There was a ~ 3‐fold increase in neutral lipid levels and LD content in the hormone‐responsive MCF7 and T‐47D breast cancer cells, and a negligible rise in HeLa cervical adenocarcinoma cells (Fig. 4B; Fig. S4A,B). DGAT inhibition consistently reduced LD levels across all cell lines (Fig. 4A,B; Fig. S4A,B). However, DHA supplementation alone, or in combination with DGAT inhibitors, caused only a weak increase in lipid ROS accumulation in A549, HeLa, and T‐47D cells, and it had no effect in MCF7 cells (Fig. 4C,D; Fig. S4C–F). Moreover, none of these cell lines showed significant changes in total ROS or cell viability following DHA and DGAT inhibitor treatments (Fig. 4E,F). The modest rise in lipid ROS levels upon DHA supplementation in A549, HeLa, and T‐47D cells was not accompanied by increased levels of cell death, which suggests that robust ferroptosis protection mechanisms neutralize lipid peroxidation in these cells.

### Context‐dependent effects of DGAT inhibition on ferroptosis sensitivity

**Fig. 5:**
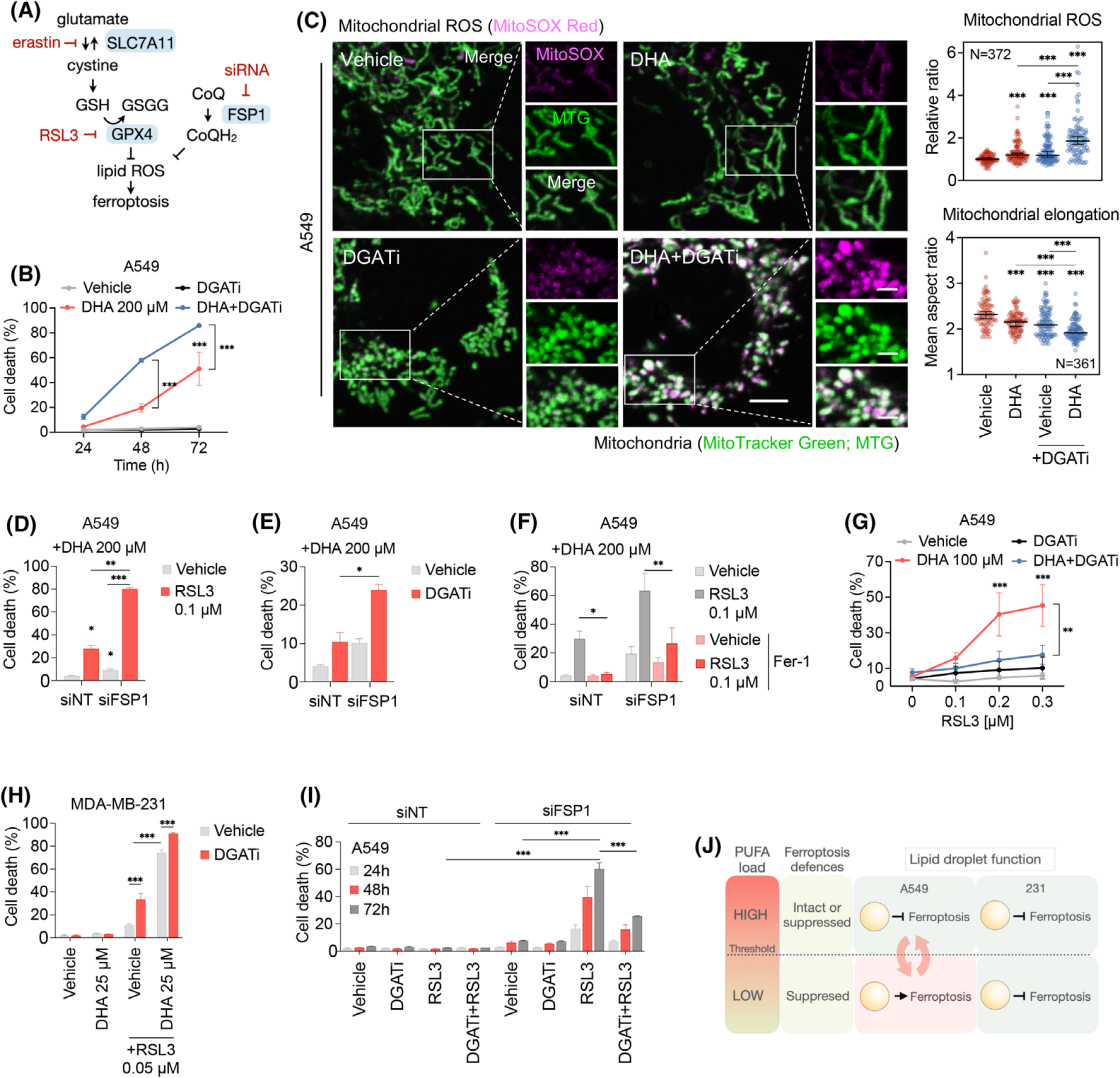
Context‐dependent roles of LD biogenesis in ferroptosis. (A) Overview of key ferroptosis surveillance mechanisms and inhibitors/siRNAs used for induction of ferroptosis. (B) Cell death (%) in A549 cells treated with 200 μm docosahexaenoic acid (DHA) for 24, 48, and 72 h, with or without 20 μm DGAT1 and 20 μm DGAT2 inhibitors (DGATi). (C) Representative confocal microscopy images of A549 cells stained with MitoSOX Red (mitochondrial ROS probe; magenta) and MitoTracker Green (mitochondria marker; green) following 24 h treatment with 200 μm DHA, with or without DGATi. Boxed insets show representative regions of a single cell displayed in separate and merged fluorescent channels. Scale bars, 5 μm and 2 μm (inset). Quantification of mitochondrial ROS (MitoSOX/MitoTracker ratio) (*n* = 372) and mitochondrial elongation (mean aspect ratio) (*n* = 361) is shown. Each point represents an individual cell. (D–F) Cell death (%) in control (siNT) and FSP1‐silenced (siFSP1) A549 cells treated with 200 μm DHA for 24 h with or without (D) 0.1 μm RSL3, (E) DGATi, and (F) 0.1 μm RSL3 and 5 μm ferrostatin‐1 (Fer‐1). (G) Cell death (%) in A549 cells treated with 100 μm DHA for 24 h, with or without DGATi, and 0.1, 0.2, or 0.3 μm RSL3. (H) Cell death (%) in MDA‐MB‐231 cells treated for 24 h with 25 μm DHA, with or without DGATi and 0.05 μm RSL3. (I) Cell death (%) in siNT and siFSP1 A549 cells treated for 24, 48, and 72 h with or without DGATi and 0.1 μm RSL3. (J) PUFA load and ferroptosis defense mechanisms (GPX4, FSP1) dictate lipid droplet roles in A549 and MDA‐MB‐231 cells. Surpassing the PUFA tolerance threshold in A549 cells induces a switch in lipid droplet function, from promoting ferroptosis to protecting against it, whereas in MDA‐MB‐231 cells lipid droplets consistently protect against ferroptosis. Cell death was determined by TMRM/YO‐PRO‐1 staining and flow cytometry. Data are presented as mean ± SEM (B, D–I) or median ± 95% CI (C) of two (B, C, F) or three (D, E, H, I) or four (G) independent biological replicates. Microscopic images are representative of three independent biological replicates. Significance was calculated by unpaired *t*‐test (B, D, E) or two‐way ANOVA with Tukey adjustment (C, F–I). *P* > 0.05 = ns; *P < 0.05; **P < 0.01; ***P < 0.001. CoQ, coenzyme Q (ubiquinone); CoQH_2_, reduced coenzyme Q (ubiquinol); FSP1, ferroptosis suppressor protein 1; GPX4, glutathione peroxidase 4; GSH, reduced glutathione; GSSG, oxidized glutathione; RSL3, Ras Selective Lethal 3; siRNA, small interfering RNA; SLC7A11, solute carrier family 7 member 11.

Our findings in ferroptosis‐resistant cell lines suggest that robust ferroptosis defense mechanisms prevent lethal lipid ROS buildup and may mask the role of LDs in modulating PUFA lipotoxicity. To uncover this potential role of LDs in A549 lung adenocarcinoma cells, we employed several strategies: altering DHA concentration, extending exposure duration, and impairing ferroptosis defense mechanisms (Fig. 5A). We first asked whether increasing the PUFA load alone could trigger cell death in A549 cells. Raising DHA levels to 200 μm and exposure time to at least 48 h resulted in significant cell death, even in the absence of ferroptosis inducers (Fig. 5B). Under these conditions, DGAT inhibition enhanced DHA‐induced cell death. Mitochondrial ROS and fragmentation were strongly elevated in DHA‐supplemented, DGAT‐inhibited A549 cells (Fig. 5C), suggesting that mitochondrial dysfunction could contribute to cell death, consistent with observations in MDA‐MB‐231 cells (Fig. 2K). When this high PUFA load was combined with RSL3 treatment (100 nm) and FSP1 knockdown, we observed a synergistic induction of ferroptotic death (Fig. 5D; Fig. S5A–D), with earlier onset and greater intensity, which was further amplified by DGAT inhibition (Fig. 5E; Fig. S5B). Ferrostatin‐1 rescued cells under these conditions, confirming ferroptotic cell death as the dominant mechanism (Fig. 5F; Fig. S5D). Together, the findings in ferroptosis‐resistant A549 cells demonstrate that a high load of exogenous DHA sensitizes these cells to ferroptosis, which is further enhanced by DGAT inhibition. Even when ferroptosis is triggered by compromising GPX4 and FSP1 function under such PUFA overload conditions, LDs retain their protective role and mitigate the synergistic amplification of ferroptosis.

We next asked whether the role of LDs could also be revealed under lower PUFA load. Treating A549 cells with progressively higher doses of RSL3 (100–300 nm) sensitized them to non‐toxic levels of DHA (100 μm), elevating both lipid ROS and cell death (Fig. 5G; Fig. S5E,F). Notably, in this context, DGAT inhibition reduced RSL3‐induced lipid peroxidation and cell death (Fig. 5G; Fig. S5E,F), reversing its earlier pro‐ferroptotic effect seen at higher PUFA levels and contrasting its role in the ferroptosis‐sensitive MDA‐MB‐231 cells. Indeed, even 50 nm RSL3 was sufficient to sensitize MDA‐MB‐231 cells to 25 μm DHA, and unlike in A549 cells, DGAT inhibition amplified this sensitisation (Fig. 5H; Fig. S5G,H). Corroborating the protective effect of DGAT inhibition observed in A549 cells, DGAT inhibition also reduced erastin‐induced ferroptosis (Fig. S5I). To determine whether exogenous PUFA is required for this protective effect, we omitted DHA and induced ferroptosis by combining RSL3 treatment with FSP1 knockdown. Under these PUFA‐poor, pro‐ferroptotic conditions, DGAT inhibition still protected A549 cells from ferroptosis (Fig. 5I). Thus, when ferroptosis is triggered by compromising ferroptosis defenses in A549 cells under basal, nutrient‐rich conditions, with or without low micromolar (< 100 μm) concentrations of exogenous DHA, LD biogenesis promotes ferroptosis.

Altogether, our findings support a model in which LDs can either promote or protect against ferroptosis depending on the context, defined by both PUFA load and the extent of ferroptosis protection (Fig. 5J). In PUFA‐tolerant and ferroptosis‐resistant A549 cells, LDs promote ferroptosis when PUFA levels are below a certain threshold and ferroptosis protection is weakened, but they become protective under very high PUFA loads, even when ferroptosis defenses remain intact. In contrast, in the PUFA‐ and ferroptosis‐sensitive MDA‐MB‐231 cells, LDs consistently protect against ferroptosis.

### Cell‐type‐specific lipid ROS localization reflects LD function in ferroptosis

**Fig. 6:**
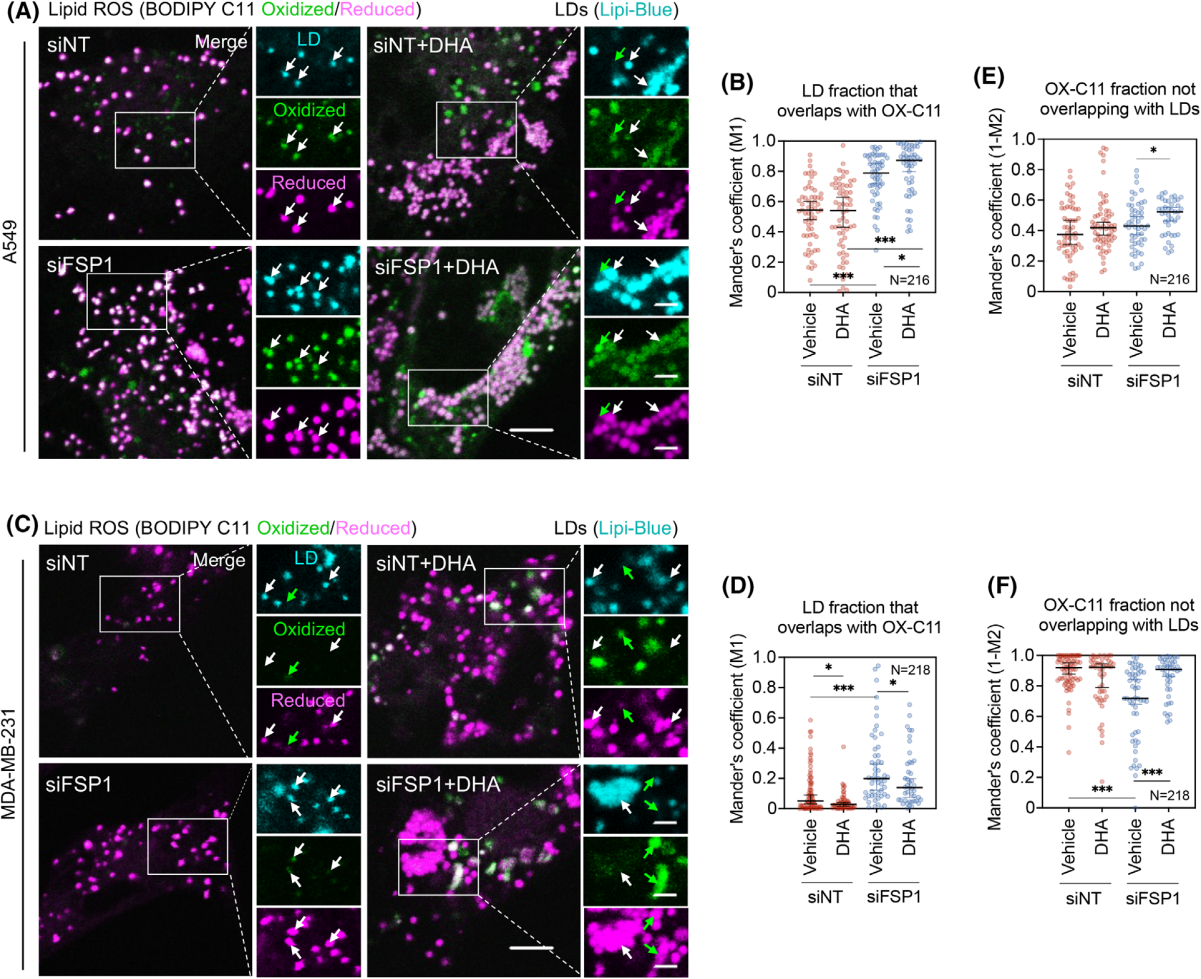
Lipid ROS localization reflects distinct roles of LDs in ferroptosis. (A) Representative confocal microscopy images of A549 cells co‐stained with the BODIPY C11 581/591 lipid peroxidation sensor (oxidized, green; reduced, magenta) and the Lipi‐Blue lipid droplet marker (cyan), following FSP1 silencing (siFSP1) and/or supplementation with 200 μm docosahexaenoic acid (DHA) for 24 h. Boxed insets show representative regions of a single cell displayed in three separate fluorescent channels. Lipid droplet localizations in all three channels are marked with white arrows. Green arrows mark oxidized probe signal that does not colocalize with lipid droplets. Scale bars, 5 μm and 2 μm (inset). (B) M1 Mander's coefficient quantifying colocalization between oxidized BODIPY C11 581/591 and Lipi‐Blue in A549 cells as in (A). Each point represents an individual cell (*n* = 216). (C) Representative confocal microscopy images of MDA‐MB‐231 cells co‐stained with the BODIPY C11 581/591 lipid peroxidation sensor (oxidized, green; reduced, magenta) and the Lipi‐Blue lipid droplet marker (cyan), following siFSP1 and/or supplementation with 50 μm DHA for 24 h. Boxed insets show representative regions of a single cell displayed in three separate fluorescent channels. Lipid droplet localizations in all three channels are marked with white arrows. Green arrows mark oxidized probe signal that does not colocalize with lipid droplets. Scale bars, 5 μm and 2 μm (inset). (D) Same analysis as in (B) performed in MDA‐MB‐231 cells upon siFSP1 and/or 50 μm DHA supplementation. Each point represents an individual cell (*n* = 218). (E) Quantification of oxidized BODIPY C11 581/591 outside lipid droplets in A549 cells as in (A). The complement of the M2 Mander's coefficient (1–M2) was calculated to determine the proportion of lipid peroxidation signal not colocalizing with lipid droplets. Each point represents an individual cell (*n* = 216). (F) Same analysis as in (E) performed in MDA‐MB‐231 cells upon siFSP1 and/or 50 μm DHA supplementation. Each point represents an individual cell (*n* = 218). Data are presented as median ± 95% CI of two independent biological replicates. Microscopic images are representative of two (A) or three (C) independent biological replicates. Significance was determined using an unpaired *t*‐test. *P* > 0.05 = ns; *P < 0.05; **P < 0.01; ***P < 0.001.

Our results in A549 cells indicate that LDs can promote ferroptosis under basal and PUFA‐poor conditions and that this activity is enhanced by RSL3 and FSP1 depletion. Lange *et al*. recently demonstrated in U2OS cells that PUFA‐loaded LDs harbor oxidized neutral lipids and initiate ferroptosis in FSP1‐deficient cells, even in the absence of GPX4 inhibitors [53]. They showed that LD‐localized FSP1 prevents neutral lipid oxidation, thereby blocking LD‐mediated promotion of ferroptosis. Inhibition of LD biogenesis under such conditions protected from ferroptosis. Consistent with this mechanism, we found that FSP1 depletion sensitized DHA‐overloaded A549 cells to ferroptosis even without RSL3 (Fig. 5E; Fig. S5B,D), suggesting that LD oxidation may also drive ferroptosis in these cells. In accordance with this idea, live‐cell confocal imaging analyses demonstrated that LDs in A549 cells substantially colocalize with the oxidized form of the BODIPY C11 lipid ROS probe, suggesting the presence of oxidized lipids within LDs even under basal conditions (Fig. 6A,B). FSP1 depletion further increased LD‐localized lipid ROS in both control and DHA‐supplemented A549 cells, supporting a protective role of FSP1 against neutral lipid oxidation. This finding also validated the use of the BODIPY C11 581/591 probe for estimating the oxidative conditions in LDs in our cellular system.

We next assessed whether FSP1 depletion might affect LD‐localized lipid ROS also in MDA‐MB‐231 cells, in which LDs protected from ferroptosis. The colocalization of the oxidized probe with LDs was minimal under both basal and DHA‐supplemented conditions in MDA‐MB‐231 cells, and was only slightly elevated upon FSP1 depletion (Fig. 6C,D), suggesting that these cells maintain a reduced LD environment even when FSP1‐mediated protection is compromised. This is in agreement with the consistently protective role of LDs against ferroptosis in these cells. Notably, DHA supplementation was not required for FSP1 depletion‐induced enhancement of LD‐localized lipid ROS in either cell line, further supporting the model of FSP1‐controlled LD‐localized lipid peroxidation. To assess whether FSP1 depletion affects the propagation of lipid oxidation from LDs to other cellular sites, a process required for ferroptotic cell death, we examined changes in lipid ROS signals outside of LDs. DHA supplementation increased the lipid ROS signal in non‐LD cellular compartments in both cell lines (Fig. 6A,C). To quantify these shifts in lipid ROS localization, we calculated the complement of the M2 Manders coefficient, representing the fraction of oxidized lipid ROS signal outside LDs. This analysis revealed that DHA‐supplementation facilitated lipid ROS accumulation in non‐LD compartments particularly in A549 cells and upon FSP1 depletion (Fig. 6E,F). Together, these data indicate that LDs in A549 cells serve as oxidative hubs promoting the propagation of lipid peroxidation under both basal and PUFA‐supplemented conditions. In contrast, LDs in MDA‐MB‐231 cells remain predominantly protective, maintaining a reduced environment, even under PUFA overload and FSP1 loss, suggesting they are not major sites of lipid oxidation in this context. In summary, our findings reveal significant cell‐type differences in the regulation of lipid ROS accumulation within LDs. These differences suggest that the role of LDs in ferroptosis is influenced by LD‐localized antioxidant mechanisms, including but not limited to FSP1.

## Discussion

The capacity of LDs to sequester, store and release various FAs is central to their function as multifaceted organelles. By controlling FA availability and distribution, LDs are essential for supporting energy production or membrane synthesis as needed [9, 15]. A less understood aspect of LD biology is their role in supplying specific FAs to maintain the diversity of membrane composition across different cellular contexts. In this study, we show that TG synthesis and LD biogenesis are critical components of the cellular response to PUFA supplementation, thereby preserving membrane and mitochondrial homeostasis, limiting lethal lipid ROS generation and preventing ferroptosis. Our data demonstrate that DGAT‐mediated LD formation maintains lipidome balance by alleviating acyl‐chain remodeling that underlies ferroptosis sensitivity. Inhibition of neutral lipid synthesis redirects excess PUFAs into membrane ether‐ and ester‐linked PLs, resulting in elevated membrane unsaturation and increased abundance of pro‐ferroptotic lipid species, such as PLs containing two polyunsaturated acyl chains [43, 62, 64]. This mechanism highlights a protective role for DGAT‐driven LD biogenesis in mitigating PUFA‐induced lipotoxicity and ferroptosis primarily by limiting PUFA incorporation into membranes. However, experiments in ferroptosis‐resistant and PUFA‐tolerant cancer cells that exhibit increased levels of ferroptosis protection reveal that the protective effect of LDs can be overridden. Notably, despite their robust ferroptosis defense mechanisms and lower cellular PUFA content [79, 82, 91], A549 lung adenocarcinoma cells accumulate lipid ROS in LDs even at basal conditions, and this increases with FSP1 depletion, supporting a mechanism of LD‐driven ferroptosis. In these cells, PUFA sequestration into LDs appears to promote lipid ROS dispersion to other compartments, shifting the role of LDs from protective to pro‐ferroptotic. Thus, our data underscore a context‐dependent role of LD biogenesis in regulating PUFA lipotoxicity and ferroptosis sensitivity.

Our findings with the ferroptosis‐ and PUFA‐sensitive MDA‐MB‐231 TNBC breast cancer cells suggest a protective role for DGAT‐mediated TG synthesis and LD biogenesis against PUFA lipotoxicity and ferroptosis. This is based on our data showing that DGAT inhibition enhances lipid ROS generation and ferroptotic cell death induced by PUFA supplementation. Our lipidomic analyses further demonstrate that LD‐driven lipidome remodeling underlies this protection. Neutral lipids stored in LDs substantially increase their unsaturation profiles upon PUFA supplementation, which points to a model of PUFA sequestration in TGs and CEs as a protective mechanism against PUFA‐induced membrane and organelle damage in ferroptosis [45, 46]. This model is supported by our data showing that the impairment of TG synthesis by DGAT inhibition leads to enrichment of PLs with PUFAs. Specifically, we found that DGAT inhibition caused PUFA redistribution across different PL classes, including ether‐ and ester‐linked PC, PE, PS, and PI species. These PLs contained either one or two polyunsaturated acyl chains, potentially contributing to ferroptosis susceptibility, as PLs from all of these four classes can be oxidized during ferroptosis [19, 21, 62, 63, 65, 66]. Additionally, DGAT inhibition in DHA‐supplemented cells caused a marked increase of PC and PE species containing two PUFA chains (PL‐PUFA_2_). PC‐PUFA_2_ species have been recently identified as drivers of ferroptosis, mediating the ferroptosis sensitizing effects of dietary PUFA‐PCs or PUFAs [43, 64]. On the other hand, PE lipids are significantly richer with PUFAs than PC species (Fig. 3H), and there is strong evidence for the involvement of both PE‐PUFA_1_ and PE‐PUFA_2_ species in ferroptosis [19, 62, 63, 95, 96]. The less abundant PS and PI species, which may also serve as substrates for peroxidation [21, 35, 65, 66], were also enriched with PUFAs following DHA supplementation, with at least one PI‐PUFA_2_ species containing AA and DHA elevated by DGAT inhibition. Nonetheless, the wide distribution of PUFA acyl chains across PL classes suggests that the general enrichment of membrane PLs could be the basis for ferroptosis sensitization in PUFA‐treated MDA‐MB‐231 cells. This finding supports a model of progressive cellular lipid peroxidation whereby the oxidation of PUFA acyl chains in various phospholipids (or other complex lipids) collectively contributes to ferroptosis [97], but depending on their localization in the cell, the local redox balance, and available repair mechanisms, they may or may not be involved in the initiation or progression of lipid peroxidation. In support of this model, the oxidation of PUFA acyl chains in neutral lipids stored in LDs can also initiate lipid peroxidation leading to ferroptosis [53]. Our results in the ferroptosis‐ and PUFA‐sensitive MDA‐MB‐231 breast cancer cells thus suggest that DGAT‐controlled sequestration of exogenous PUFAs in TGs protects against ferroptosis by broadly reducing PUFA enrichment across phospholipid classes, limiting both high‐ and low‐abundance ferroptosis drivers, including PL‐PUFA_2_ species.

Our data comparing LD function in ferroptosis‐resistant and ‐sensitive cancer cell lines point at several mechanisms that contribute to their differential PUFA tolerance and the context‐dependent role of LDs in ferroptosis regulation. The basis of these differences in ferroptosis sensitivity lies in multiple factors that converge at the metabolic, redox and microenvironmental levels. The ferroptosis‐resistant cancer cells used in this study display strong intrinsic ferroptosis protection and reduced membrane PUFA content (Table S1) [29, 63, 74, 75, 79, 82, 90]. Elevated *de novo* lipogenesis, which increases the incorporation of saturated and monounsaturated fatty acids into membrane phospholipids, together with robust antioxidant defenses, underlies their high PUFA tolerance and ferroptosis resistance [36, 76, 79]. In A549 lung adenocarcinoma cells, antioxidant transcriptional programs controlled by nuclear factor erythroid 2‐related factor (Nrf2) are constitutively active due to a mutation in the Nrf2 negative regulator Kelch‐like ECH‐associated protein 1 (KEAP1) [86, 92, 93]. KEAP1 dysfunction in A549 lung cancer cells leads to increased FSP1 expression [90, 91], effectively limiting lipid peroxidation. A549 cells also restrict membrane PUFA availability via their low long‐chain acyl‐CoA synthetase 4 (ACSL4) expression, which favors PUFA incorporation into membranes, and elevated ACSL3 expression, which prefers monounsaturated fatty acids [81, 82]. Our data suggest that these potent ferroptosis protective mechanisms and lipid metabolic reprogramming limit the capacity of LDs in A549 cells to influence ferroptosis sensitivity through PUFA trafficking. Consequently, to reveal a role for LDs in these cells, it was necessary to overload them with exogenous PUFAs and/or to impair GPX4 and FSP1 activity (Fig. 5J). Under moderate PUFA loading and when ferroptosis protection was weakened, DGAT‐mediated LD biogenesis sensitized cells to ferroptosis; however, LDs became protective at very high PUFA loads, even when ferroptosis defenses remained intact. In contrast, in the PUFA‐rich and ferroptosis‐sensitive MDA‐MB‐231 cells, LDs consistently protected against ferroptosis.

Our data further suggest that cell‐line‐specific differences in LD‐localized prevention of lipid peroxidation, mediated by FSP1 [53] and other yet unknown mechanisms, can strongly influence LD function in ferroptosis modulation. Our findings using the BODIPY‐C11 lipid ROS probe, which serves as an estimate of the redox state of its environment [98], point to differences in the LD oxidative state between MDA‐MB‐231 and A549 cells. In MDA‐MB‐231 cells, LDs primarily contained the reduced probe under basal conditions, and this was largely unaffected by DHA supplementation or FSP1 knockdown. In contrast, LDs in A549 cells accumulated the oxidized probe even under basal conditions, and following FSP1 knockdown, nearly all LDs in these cells became oxidized. This suggests the possibility that ferroptosis‐sensitive MDA‐MB‐231 cells harbor LDs that are more resistant to oxidation, which is consistent with the model of PUFA sequestration in neutral lipids as a protective and predominant mechanism in these cells. Consequently, DGAT inhibition, which redirects PUFAs to membranes, becomes detrimental to these cells. Conversely, the predominantly oxidized LDs in the ferroptosis‐resistant A549 cells may contribute to their resistance by limiting membrane lipid peroxidation even under basal conditions. However, when LD oxidation is further enhanced by FSP1 deficiency, it could promote the spread of lipid peroxidation, as observed in PUFA‐overloaded U2OS cells [53]. Consistent with this LD‐driven ferroptosis, our data suggest that DHA sequestration within LDs combined with FSP1 deficiency facilitates lipid ROS dispersion to other cellular compartments. This may explain why DGAT inhibition in A549 cells protects against ferroptosis induced by the combination of GPX4 inhibition, FSP1 knockdown and low to moderate PUFA doses.

However, in A549 cells exposed to very high PUFA loads, the role of LD biogenesis shifted from sensitization to protection from DHA‐induced injury. This suggests that LDs serve multiple functions in ferroptosis, with their relative importance varying depending on cellular conditions. Under excessive PUFA loads, the protective effect of PUFA sequestration in LDs, which includes prevention of membrane PUFA enrichment and mitochondrial dysfunction, may outweigh the pro‐ferroptotic influence of LD neutral lipid peroxidation. Additionally, very high PUFA levels likely induce various lipotoxic effects beyond ferroptosis, aligning with the broader role of LDs as primary fatty acid sequestration sites that protect against lipotoxic stress. Therefore, our findings underscore a context‐dependent role of LD biogenesis in the control of PUFA lipotoxicity and ferroptosis sensitivity depending on the metabolic and oxidative state of the cell.

Our results in DHA‐supplemented MDA‐MB‐231 and A549 cells indicate that impaired TG synthesis redirects PUFAs toward mitochondria, leading to mitochondrial dysfunction. In MDA‐MB‐231 cells, the observed increase in polyunsaturated acylcarnitine levels suggests that this redirection ultimately exceeds mitochondrial capacity, thereby preventing efficient acylcarnitine import. These findings align with previous reports showing that DGAT inhibition causes mitochondrial damage due to acylcarnitine accumulation following FA overload triggered by starvation‐induced autophagy [59]. Intact DGAT activity also supports mitochondrial homeostasis by regulating mitochondrial ROS levels and mitophagy, which is essential for clearing damaged mitochondria [99, 100]. While PUFA supplementation alone caused only a modest increase in mitochondrial ROS in both cell lines, this effect was significantly amplified upon DGAT inhibition. The resulting ROS production likely contributes to ferroptosis, as shown in cells supplemented with PC‐PUFA_2_ [43]. These lipids, which were upregulated by DHA supplementation and DGAT inhibition in our system, were recently shown to promote mitochondrial ROS generation and ferroptosis initiation through interactions with ETC complex I [43]. Concurrently, we observed an increase in mitochondrial fragmentation, an indicator of mitochondrial dysfunction recently implicated in ferroptosis [60, 61]. Specifically, the mitochondrial fission regulator dynamin‐related protein 1 (Drp1) is activated during ferroptosis and promotes cell death execution through mitochondrial fragmentation [61]. Taken together, our data suggest that DGAT‐mediated LD biogenesis supports mitochondrial homeostasis in DHA‐supplemented cells, potentially preventing both ferroptosis and non‐ferroptotic mitochondrial lipotoxicity [43, 61, 101, 102].

Beyond ferroptosis, our data support the idea that cells tightly regulate membrane unsaturation to maintain a narrow range of physicochemical properties essential for membrane function and cellular fitness [68]. PUFA incorporation into membrane phospholipids increases membrane unsaturation, which can disrupt cellular homeostasis by altering key biophysical properties, such as membrane packing, bending, and fluidity [17, 103]. To counteract this, cells upregulate saturated PLs and cholesterol in membranes [68]. In our study, we observed a clear upregulation of saturated PLs and sphingolipids in DHA‐supplemented cells, which likely helps maintain membrane rigidity following PUFA enrichment. This adaptive response may explain the need for a substantial plasticity of the LD lipidome, which, unlike cellular membranes, can significantly alter its unsaturation profile upon PUFA supplementation. It is thus possible that PUFA‐induced membrane perturbations drive PUFA sequestration into LDs primarily to preserve membrane homeostasis, while also reducing the likelihood of membrane lipid peroxidation. Therefore, the sequestration of PUFAs into neutral lipids stored in LDs provides multiple benefits for the cell, including maintaining membrane function by regulating unsaturation levels and reducing ferroptosis susceptibility by limiting the availability of peroxidation‐prone acyl chains in membranes.

This study identifies LDs as multifunctional hubs controlling ferroptosis through the regulation of membrane unsaturation, lipid peroxidation, and mitochondrial integrity in human cancer cells. Which of the two DGAT enzymes is critical for the observed ferroptosis modulation in our system remains unknown. DGAT1, an ER‐resident transmembrane protein, catalyzes TG synthesis within the ER bilayer, whereas DGAT2 can relocalize from the ER to LDs to mediate LD‐localized neutral lipid synthesis [104, 105, 106, 107]. Because these enzymes compensate for one another, dual inhibition is necessary to fully block TG production and LD biogenesis. How DGAT inhibition redirects PUFAs toward phospholipids is also unresolved. The accumulation of fatty acids and DGs likely promotes phospholipid synthesis by increasing substrate availability for enzymes in the glycerol‐3‐phosphate pathway, such as glycerol‐3‐phosphate acyltransferases (GPATs) and acylglycerol‐3‐phosphate acyltransferases (AGPATs) [48, 108, 109]. Elevated free fatty acids may further promote phospholipid remodeling through the Lands' cycle, mediated by phospholipases A_2_ and lysophospholipid acyltransferases (LPCATs) [110]. Several of these lipid metabolic enzymes, including ACSL4 and LPCAT3, are established ferroptosis regulators [63, 111]. DGAT inhibition and the resulting accumulation of DG, phosphatidic acid and free fatty acids, may also disrupt ER membrane homeostasis and the function of various proteins involved in LD formation. The seipin‐LDAF1 complex and FIT proteins bind TGs within the ER membrane and depend on their presence to regulate LD nucleation and growth [112, 113, 114, 115]. TG insufficiency and DG accumulation may thus stall seipin‐LDAF1 complexes and disrupt the localization and function of many LD‐associated proteins that bind to nascent or growing LDs, including the perilipins [116, 117]. Finally, a limitation of this study is that LD function was inferred indirectly by studying the impact of LD loss. Future work should explore how LD turnover and interactions with other organelles contribute to ferroptosis regulation, and how LD breakdown pathways complement FSP1 lipid quality control at the LD surface.

This work has important implications for basic cell biology and cancer research, though it remains unclear how broadly these mechanisms apply in disease and therapeutic settings. Targeting ferroptosis is a promising therapeutic strategy in particular in therapy‐resistant and metastatic cancers, which show increased ferroptosis susceptibility [39, 118, 119, 120, 121]. Recent work has demonstrated that FSP1 inhibition can limit melanoma growth in lymph nodes [122] and suppress lung adenocarcinoma tumor growth *in vivo*, even when GPX4 is intact [91]. PUFA‐rich dietary interventions together with DGAT inhibition have already shown some therapeutic potential [42]. However, the opposing roles of LDs in ferroptosis regulation in different cancer models, and in particular the context‐dependent switch in LD function revealed in A549 cells, suggest that therapeutic interventions targeting lipid metabolism must be carefully tailored to match specific cancer types and their metabolic/redox characteristics. LD‐controlled PUFA metabolism and ferroptosis modulation has potential therapeutic implications also beyond cancer. In neurodegeneration, promoting lipid storage in glial cells may be neuroprotective, as sequestering oxidation‐prone PUFAs into TG‐rich LDs limits lipid peroxidation and oxidative damage in neurons [45, 48, 123, 124, 125, 126, 127]. It will be important for future studies to see how widespread is the protective versus pro‐ferroptotic role of LDs in cancer, neurodegeneration, and other pathologies.

## Materials and methods

### Chemicals

RPMI‐1640 Medium (30–3001) and Eagle's Minimum Essential Medium (EMEM) (30–2003) were obtained from ATCC (USA). Dulbecco's Modified Eagle's Medium/Nutrient Mixture F‐12 (DMEM/F‐12, 11 330 057), DMEM with high glucose and GlutaMAX supplement (DMEM/GlutaMAX, 61 965–026), Dulbecco's Phosphate‐Buffered Saline (DPBS, 14190169), Fetal Bovine Serum (FBS, 10500064), Hanks' Balanced Salt Solution (HBSS, 14025050), Hoechst 33342 (H3570), MitoTracker Green FM (M7514), MitoSOX Red (M36008), RPMI‐1640 Medium without phenol red (11835063), TrypLE Select Enzyme (12563–029), L‐Glutamine (25030081), Lipofectamine RNAiMAX Transfection Reagent (13778100) and Opti‐MEM (11058021) were purchased from Thermo Fisher Scientific (USA). Human FSP1‐targeting siRNAs and the AllStars Negative Control siRNA were from Qiagen (Germany). Docosahexaenoic acid (90310), oleic acid (90260), erastin (E7781), ferrostatin‐1 (17729), MCC950 (17510), necrostatin‐2 (Nec‐1S, 20 924), olaparib (10621), (1S,3R)‐RSL3 (19288) were obtained from Cayman Chemical (USA). BODIPY 493/503 (D3922), CM‐H2DCFDA (C6827), BODIPY 581/591 C11 Lipid Peroxidation Sensor (D3861), Nile Red (N1142), Tetramethylrhodamine (TMRM) (T668), and YO‐PRO‐1 Iodide (Y3603) were from Thermo Fisher Scientific (USA). 7‐aminoactinomycin D (7‐AAD) (A9400), Insulin solution human (I9278), T863 (SML0539), PF‐06424439 (PZ0233), essentially fatty acid‐free (A7511)/fatty acid‐free (A8806) bovine serum albumin (BSA) were purchased from Sigma‐Aldrich (USA). Liproxstatin‐1 (S7699), necrostatin‐1 (S8037), z‐VAD‐fmk (S7023) were from SelleckChem (USA). Lipi‐Blue (LD01) was purchased from Dojindo (Japan). DTT (R0862), Novex Tris‐Glycine SDS 2× Sample Buffer (LC2676), EDTA‐Free 100× Halt Protease Inhibitor Cocktail (78425), and Pierce 660 nm Protein Assay Kit (22662) were obtained from Thermo Fisher Scientific (USA). For Western blot (WB) analysis, WB‐grade BSA from Sigma‐Aldrich (USA) (A7030) was used. Nitrocellulose membrane (71224.01) was from SERVA (Germany), and Western Blocking Reagent (WBR) (11921673001) and Lumi‐Light Western Blotting Substrate (12015196001) were from Roche Applied Science (Germany). β‐actin primary antibodies (NB600‐532) were from Novus Biologicals (UK), FSP1 primary antibodies (sc‐37 712) were from Santa Cruz Biotechnology (USA). Horseradish peroxidase‐conjugated Secondary Antibodies were from Jackson ImmunoResearch Laboratories (USA). All other chemicals were of at least analytical grade and purchased from Sigma‐Aldrich (USA) or Serva (Germany).

### Cell culture conditions and treatments

The human cancer cell lines MDA‐MB‐231 (RRID: CVCL_0062), T‐47D (RRID: CVCL_0553), MCF‐7 (RRID: CVCL_0031), A‐549 (RRID: CVCL_0023), and HeLa (RRID: CVCL_0030) were obtained from the American Type Culture Collection (ATCC, USA). Cell lines were cultured in the following media, all supplemented with 10% FBS: RPMI‐1640 for MDA‐MB‐231 cells, DMEM/F‐12 for A549 cells, DMEM/GlutaMAX for HeLa cells, RPMI‐1640 supplemented with 0.2 U·mL^−1^ human insulin for T‐47D cells, and EMEM supplemented with 0.01 mg·mL^−1^ human insulin for MCF‐7 cells. All cells were maintained at 37 °C in a humidified atmosphere with 5% CO_2_, used at early passages, and passaged no more than 10 times. Mycoplasma contamination was routinely tested.

Fatty acids were stored as aliquoted stock solutions (at 10–50 mm concentrations) in absolute ethanol under argon at −80 °C. Prior to treatment, stock solutions were diluted in cell‐line‐specific serum‐containing culture medium and incubated for 1 h at room temperature with gentle mixing to allow for efficient complexation of fatty acids with serum albumin. The final ethanol concentrations were kept between 0.167 and 0.67% to avoid cytotoxicity and appropriate vehicle controls were included in every experiment. DGAT1 and DGAT2 inhibitors (T863 and PF‐06424439, respectively) were added to cells 2 h before treatments with fatty acids, ferrostatin‐1, RSL3, or erastin and were present in the medium for the duration of the treatments. Appropriate vehicle controls (e.g., ethanol or DMSO) were included in all experiments to account for solvent effects.

### Gene silencing using small‐interfering RNA

Reverse transfection was carried out in either 24‐well, 6‐well, or 4‐well plates using the following seeding densities: 3 × 10^4^ or 5 × 10^4^ A549 cells/well and 6 × 10^4^ MDA‐MB‐231 cells per well in 4‐ and 24‐well plates; 2.5 × 10^5^ A549 cells per well and 3 × 10^5^ MDA‐MB‐231 cells per well in 6‐well plates. Cells were transfected with an equimolar mixture of four FSP1‐specific siRNAs (each at 5 nm, for a total siRNA concentration of 20 nm). siRNA sequences are listed in Table S2. AllStars Negative Control siRNA (20 nm) was used as a non‐targeting siRNA control in all knockdown experiments. Transfection complexes were prepared using Lipofectamine RNAiMAX Transfection Reagent (1 μL per well for 4‐ and 24‐well plates; and 7.5 μL per well for 6‐well plates) and Opti‐MEM medium, following the manufacturer's instructions.

### Flow cytometry analysis

For all flow cytometry assays, cells were seeded into 24‐well culture plates at a density of 3 × 10^4^ or 6 × 10^4^ cells per well and incubated for 24 h prior to treatment. Unless otherwise specified, cells were harvested after treatment, transferred to FACS tubes along with the culture supernatant, and centrifuged at 300 × ***g*** for 10 min. For neutral lipid analysis, cells were stained with 1 μg·mL^−1^ Nile Red in DPBS for 10 min in the dark and analyzed using an excitation laser of 488 nm and an emission filter of 530/30 nm (FL‐1 channel) for Nile Red. At least 20 000 events were collected per sample using the Cell Quest software (Becton Dickinson, USA). For cell death assays, cells were stained with 25 nm TMRM in DPBS for 15 min in the dark, followed by staining with 50 nm YO‐PRO‐1 for an additional 10 min in the dark. Samples were diluted in DPBS containing 0.1% fatty acid‐free BSA and analyzed using an excitation of 488 nm, an FL‐1 emission filter of 530/30 nm for YO‐PRO‐1 and a 650 long‐pass FL‐3 filter (650LP) for TMRM. YO‐PRO‐1^+^/TMRM^−^ cells were gated as dead. At least 30 000 events were collected per sample. For lipid peroxidation analyses, attached cells were stained prior to treatment with 1 μm BODIPY 581/591 C11 in HBSS at 37 °C for 30 min. Following treatment, cells were harvested, centrifuged, resuspended in HBSS and analyzed using an excitation of 488 nm, an emission filter of 530/30 nm (FL‐1 channel) for the oxidized form of BODIPY C11 and FL‐3 filter (650LP) for the reduced form of BODIPY C11. At least 20 000 events were collected for each sample. For total ROS and cell death analysis, cells were stained with 1 μm CM‐H2DCFDA in HBSS for 30 min, followed by a 2 h incubation in phenol red‐free culture medium. Subsequently, cells were harvested, stained with 5 μg·mL^−1^ 7‐AAD for 10 min and analyzed by flow cytometry. Samples were excited at 488 nm and the emission fluorescence signal recorded with an 530/30 nm FL‐1 filter for CM‐H2DCFDA and an 650LP FL‐3 filter for 7‐AAD. ROS levels were determined in live cells (7‐AAD^−^). At least 20 000 events were collected for each sample. For all flow cytometry assays, at least 2 independent biological replicates were performed. Data were visualized and gated using FSC/SSC, FL1/FL3 plots and histograms (cell count/signal). Geometric means were used to calculate population averages for neutral lipid accumulation and ROS levels; cellular lipid peroxidation levels were visualized using histograms and calculated as ratios of geometric means of the FL‐1 (oxidized dye) and FL‐3 (non‐oxidized dye) signals; percentages of YO‐PRO‐1^+^/TMRM^−^ gated cells within the total population of cells were used for calculations of cell death levels.

### Live‐cell imaging

Cells were seeded into 4‐well glass bottom culture plates (Greiner Bio‐One, 627 870) at a density of 3 × 10^4^ or 6 × 10^4^ cells per well and incubated for 24 h. For lipid droplets and nuclei visualization, cells were washed with warm DPBS then stained for 15 min at 37 °C with 1 μm BODIPY 439/503 and 1 μg·mL^−1^ Hoechst 33342 in culture medium without FBS, washed with warm DPBS and imaged. For visualization of mitochondria and mitochondrial ROS, cells were washed with warm HBSS then stained for 30 min at 37 °C with 100 nm MitoTracker Green and 1 μm MitoSOX Red in HBSS, washed with warm HBSS and imaged. For lipid peroxidation analysis, cells were washed with warm PBS and stained with 1 μm BODIPY 581/591 C11 in HBSS at 37 °C for 30 min prior to treatment. For lipid droplet and BODIPY C11 colocalization imaging, cells were stained with BODIPY 581/591 C11 prior to treatment as described above. Following treatment, cells were washed with warm DPBS, stained with 0.1 μm Lipi‐Blue in culture medium without FBS for 15 min at 37 °C, washed with warm DPBS and imaged. Imaging was performed using a confocal laser scanning microscope (LSM 710; Carl Zeiss, Germany) with a stage‐top CO_2_ incubation system (Tokai Hit, Japan). Images were processed and analyzed with ZEN (Carl Zeiss, Germany) and imagej (National Institute of Health, USA) software.

### Image analysis

All image analysis was performed using Fiji [128] on at least 50 cells per sample. For mitochondrial ROS quantification, background‐subtracted MitoSOX Red and MitoTracker Green fluorescence intensities were measured from split channels. The ratios (MitoSOX Red/MitoTracker Green) were calculated per cell and normalized to the median value of the corresponding biological replicate. Quantitative analysis of mitochondrial morphology was performed using Mitochondria Analyzer plugin [129], following the developer's instructions. For colocalization quantification, confocal images were split into individual channels and processed side‐by‐side with the JaCoP plugin, following the developer's instructions. Mander's coefficients (M1 and M2) were calculated individually for each cell using manual thresholding.

### Western blot analysis

Cells were seeded into 6‐well culture plates at a density of 1.5 × 10^5^ or 3 × 10^5^ cells per well for 24 h. The medium was then replaced with fresh culture medium and cells were incubated for an additional 24 h. Cell lysates were prepared by washing cells with ice‐cold DPBS and scraped into Tris‐glycine SDS 2× sample buffer with 800 mm DTT and Halt Protease Inhibitor Cocktail. Lysates were incubated at 95 °C for 10 min and either stored on ice for immediate use or frozen at −80 °C for later analysis. Protein concentration was determined using the Pierce 660 nm Protein Assay Kit according to the manufacturer's instruction. Equal amounts of protein (10 μg per sample) were loaded onto 10% SDS/PAGE gels and separated by electrophoresis. Proteins were then transferred onto nitrocellulose membranes using a wet western blot transfer system. Membranes were blocked for 1 h at room temperature in 1% Western Blocking Reagent (WBR) in TBS and incubated overnight at 4 °C with primary antibodies diluted in 0.5% WBR in 0.1% TBS‐T (TBS with 0.1% Tween‐20). After three washes with TBS‐T, membranes were incubated with horseradish‐peroxidase‐labeled secondary antibodies diluted 1 : 10000 in 0.5% WBR in TBS‐T for 1 h at room temperature. Membranes were washed three times in TBS‐T, signal detection was performed using Lumi‐Light Western Blotting Substrate, and chemiluminescent signals were visualized using a Gel Doc XR system (Bio‐Rad, USA). Densitometric analysis of the blots was performed using Fiji. Band intensities were quantified by measuring the area of each peak, with background correction applied to all measurements. Target protein levels were normalized to the corresponding β‐actin loading control. Quantification was based on three independent experiments.

### Sample preparation for untargeted Lipidomics

MDA‐MB‐231 cells were seeded into 6‐well culture plates at a density of 3 × 10^5^ cells per well and allowed to adhere for 24 h. Cells were then pretreated for 2 h with or without 20 μm DGAT1 and 20 μm DGAT2 inhibitors (DGATi), followed by 25 μm docosahexaenoic acid (DHA) supplementation with or without DGATi for 1, 4, and 24 h. After incubation, cells were washed twice with DPBS and harvested by scraping in 550 μL of LC/MS‐grade 55 : 45 methanol:water. Samples were stored at −80 °C until analysis. Protein content was determined in each sample using the Pierce 660 nm Protein Assay Kit following the manufacturer's instructions and used to normalize lipid signals. The experiment included three independent biological replicates. LC/MS‐grade methanol (8402–2500) was from Avantor and LC/MS‐grade water (39253‐1 L) was from Honeywell Chemicals.

### Lipid extraction and untargeted LC/MS–MS Lipidomics

To extract complex lipids, we processed the samples using the LIMeX LC–MS workflow (LIpids, Metabolites, and eXposome compounds). Metabolites were extracted with a biphasic solvent system consisting of cold methanol, methyl tert‐butyl ether, and 10% methanol [130]. An aliquot of the upper organic phase was collected, evaporated, and resuspended in methanol containing the internal standard [12‐[(cyclohexylamino)carbonyl]amino]‐dodecanoic acid (CUDA). The samples were then analyzed using lipidomics platforms in both positive and negative ion modes. For untargeted lipidomics, the LC–MS systems consisted of a Vanquish UHPLC System (Thermo Fisher Scientific, Bremen, Germany) coupled to a Q Exactive Plus mass spectrometer (Thermo Fisher Scientific, Bremen, Germany). We separated the lipids on an ACQUITY Premier BEH C18 column (50 × 2.1 mm; 1.7 μm) with VanGuard FIT (5 × 2.1 mm; 1.7 μm) and detected them using positive and negative electrospray ionization (ESI). Raw data were processed using MS‐DIAL 4.94 [131] with an in‐house retention time–m/z library and MS/MS libraries from public and commercial sources (MassBank, MoNA, NIST23). Data were filtered using blank samples, serial dilution samples, and quality control (QC) pool samples with a relative standard deviation (RSD) < 30%. Normalization was performed using the LOESS approach, based on QC pool samples injected regularly between every 10 actual samples. Samples were randomized across the platform run. Metabolites were annotated according to the LIPID MAPS classification system and the shorthand notation for lipid structures [132]. All analytes in MS‐DIAL were manually curated.

### Lipidomic data analysis

Data analysis was performed using protein‐normalized signal intensities (arb. units/μg protein). Outliers within biological triplicates were identified by calculating the coefficient of variation (CV); values with a CV greater than 30% were considered outliers and removed. Missing values were imputed by averaging the remaining two biological replicates. Lipids in individual samples that still exhibited a CV greater than 30% after this step were excluded from further analysis. For heatmap analysis, lipids were sorted by increasing carbon number and degree of unsaturation (number of double bonds). Lipid signal intensities were normalized and plotted on a heatmap without data clustering. For volcano plot analysis, lipid signal intensities were log_2_‐transformed, and multiple unpaired *t*‐tests (*P* < 0.05) were performed. To analyze relative lipid class abundance, individual lipids were grouped according to their respective lipid classes, and the summed intensities of all lipids within each lipid class were calculated for each sample. To determine the relative abundances of SFA‐, MUFA‐, and PUFA‐containing TGs and PLs, lipids were grouped based on their acyl‐chain unsaturation. The relative abundances of all lipids within each group were summed to obtain a cumulative value, which was used to calculate the proportion of each unsaturation group relative to all TGs and PLs. The unsaturation index was calculated for each lipid by weighing the total number of double bonds by the relative abundance of that lipid within its lipid class. The individual indexes were summed for each replicate to generate a class‐level unsaturation index. For LORA, query lists were generated based on multiple unpaired *t*‐tests (*P* < 0.1) on log_2_‐transformed lipidomic data, selecting significantly upregulated lipids. Enrichment analysis was performed using the LORA online application [69]. Principal component analysis (PCA) was conducted using Metaboanalyst 5.0 to assess the distribution and consistency of the lipidomic data. graphpad prism 10.3.1 (version 10, GraphPad Software Inc., USA) was used for statistical analysis, data transformation, and plotting of the data.

### Statistical analysis

All data were plotted and statistical analyses performed using graphpad prism 10.3.1 (version 10, GraphPad Software Inc., USA). Data are reported as mean ± SEM, except for microscopy quantifications, which are reported as median ± 95% CI. For each experiment, the number of replicates is indicated in the corresponding figure legends. Statistical significance was determined using two‐tailed unpaired *t*‐tests or two‐way ANOVA, followed by Tukey's multiple comparison tests. Unless otherwise stated, *P* < 0.05 was considered statistically significant.

## Author contributions

AK and LP performed most experiments, analyzed data, prepared figures and wrote manuscript draft; ŠK, EJJ prepared samples for mass spectrometry analysis, assisted with flow cytometry and microscopy, and analyzed data; NF performed some of the flow cytometry experiments; CPK performed data analysis; KB and OK performed mass spectrometry analyses, lipidomic data analysis, assisted with experimental design, contributed ideas and revised the manuscript; MF and MW contributed ideas, lipidomics expertise and revised the manuscript. TP conceptualized the study, analyzed data, prepared figures, and revised the manuscript.

## Conflicts of interest

The authors declare no conflicts of interest.

## Supporting information


**Fig. S1.** Oleic acid induces DGAT‐mediated LD accumulation but not ferroptosis.
**Fig. S2.** DHA and DGATi‐induced changes in LD‐stored neutral lipids.
**Fig. S3.** DHA and DGATi‐induced changes in PL acyl‐chain composition.
**Fig. S4.** DHA‐ and DGATi‐induced changes in LD and lipid ROS levels in ferroptosis‐resistant cells.
**Fig. S5.** DGAT inhibition protects or promotes ferroptosis depending on the context.
**Table S1.** Features of cancer cell lines used in this study.
**Table S2.** List of siRNA sequences used in this study.

## Data Availability

All data necessary to support the conclusions of this study are included in the main text, figures and Supporting Information. Lipidomics data are openly available at the DRYAD repository (http://doi.org/10.5061/dryad.2bvq83c4h). Additional data are available from the corresponding author on reasonable request.
