# Supplementary material for: Opposing roles of DGAT‐mediated lipid droplet biogenesis in the regulation of ferroptosis sensitivity
Source: FEBS J. 2026 Feb 23;293(15):4508–32. doi: 10.1111/febs.70467 (PMC13440611; doi:10.1111/febs.70467)
Supplement: Supplementary file 1 [file FEBS-293-4508-s001.pdf]

Supporting Information

Opposing Roles of DGAT-mediated Lipid Droplet Biogenesis in the Regulation of Ferroptosis Sensitivity

Ana Kump[#1,2], Leja Perne[#1,2], Špela Koren[1,2], Eva Jarc Jovičić[1], Nastja Feguš[1], Carina Pinto Kozmus[1], Michele Wölk[3], Kristyna Brejchova[4], Maria Fedorova[3], Ondrej Kuda[4], Toni Petan*[1]

[1]Department of Molecular and Biomedical Sciences, Jožef Stefan Institute, Ljubljana, Slovenia
[2]Jožef Stefan International Postgraduate School, Ljubljana, Slovenia
[3]Center of Membrane Biochemistry and Lipid Research, University Hospital and Faculty of Medicine Carl Gustav Carus of TU Dresden, Dresden, Germany
[4]Metabolism of Bioactive Lipids, Institute of Physiology of the Czech Academy of Sciences, Prague, Czechia

[#]Authors contributed equally

*Correspondence
Toni Petan, Jožef Stefan Institute, Ljubljana, Slovenia
Tel: +386 1 477 3713
E-mail: toni.petan@ijs.si

Contents

Fig. S1. Oleic acid induces DGAT-mediated LD accumulation but not ferroptosis.

Fig. S2. DHA and DGATi-induced changes in LD-stored neutral lipids.

Fig. S3. DHA and DGATi-induced changes in PL acyl-chain composition.

Fig. S4. DHA- and DGATi-induced changes in LD and lipid ROS levels in ferroptosis-resistant cells.

Fig. S5. DGAT inhibition protects or promotes ferroptosis depending on the context.

Table S1. Features of cancer cell lines used in this study.

Table S2. List of siRNA sequences used in this study.

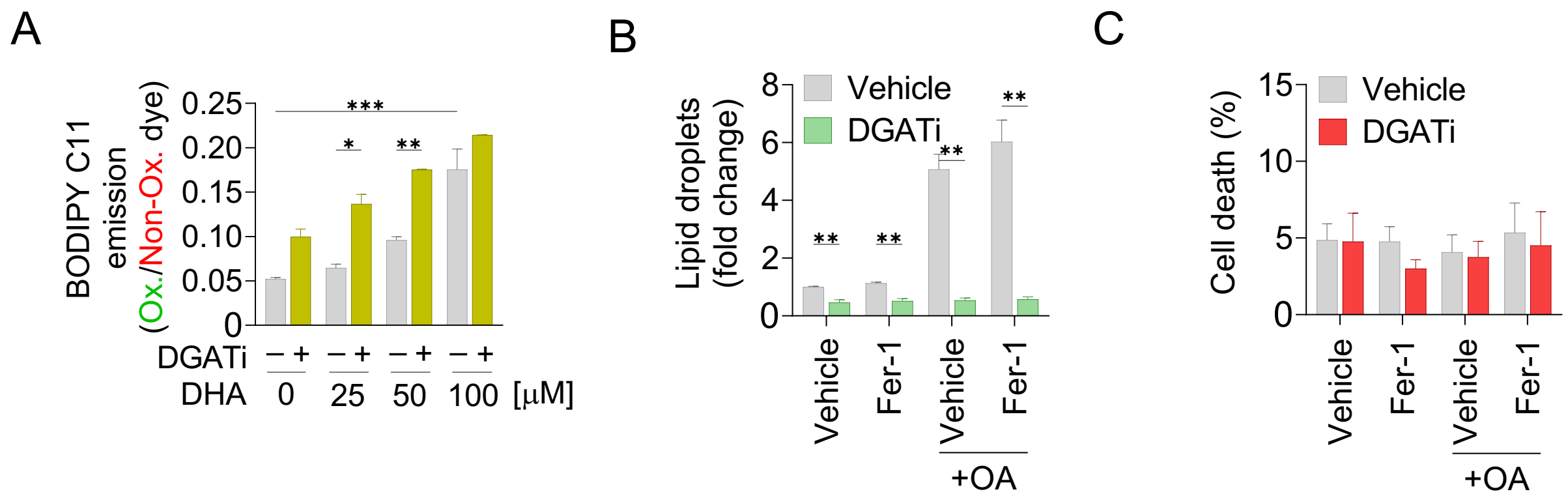

Figure S1. Oleic acid induces DGAT-mediated LD accumulation but not ferroptosis.

(A) Lipid peroxidation assessed by BODIPY C11 581/591 probe using flow cytometry in MDA-MB-231 cells treated with 25–100 μM docosahexaenoic acid (DHA) for 24 h, with or without 20 μM DGAT1 and 20 μM DGAT2 inhibitors (DGATi).

(B) Lipid droplet levels measured by Nile Red staining in MDA-MB-231 cells treated with 100 μM oleic acid (OA) and/or 5 μM ferrostatin (Fer-1) for 24 h, with or without DGATi.

(C) Cell death (%) determined by TMRM/YO-PRO-1 staining in MDA-MB-231 cells following the treatment described in (B).

All data are presented as mean ± SEM of two (A) or three (B, C) independent biological replicates (significance was calculated by two-way ANOVA with Tukey adjustment). $p > 0.05$ = ns; *$p < 0.05$; **$p < 0.01$; ***$p < 0.001$.

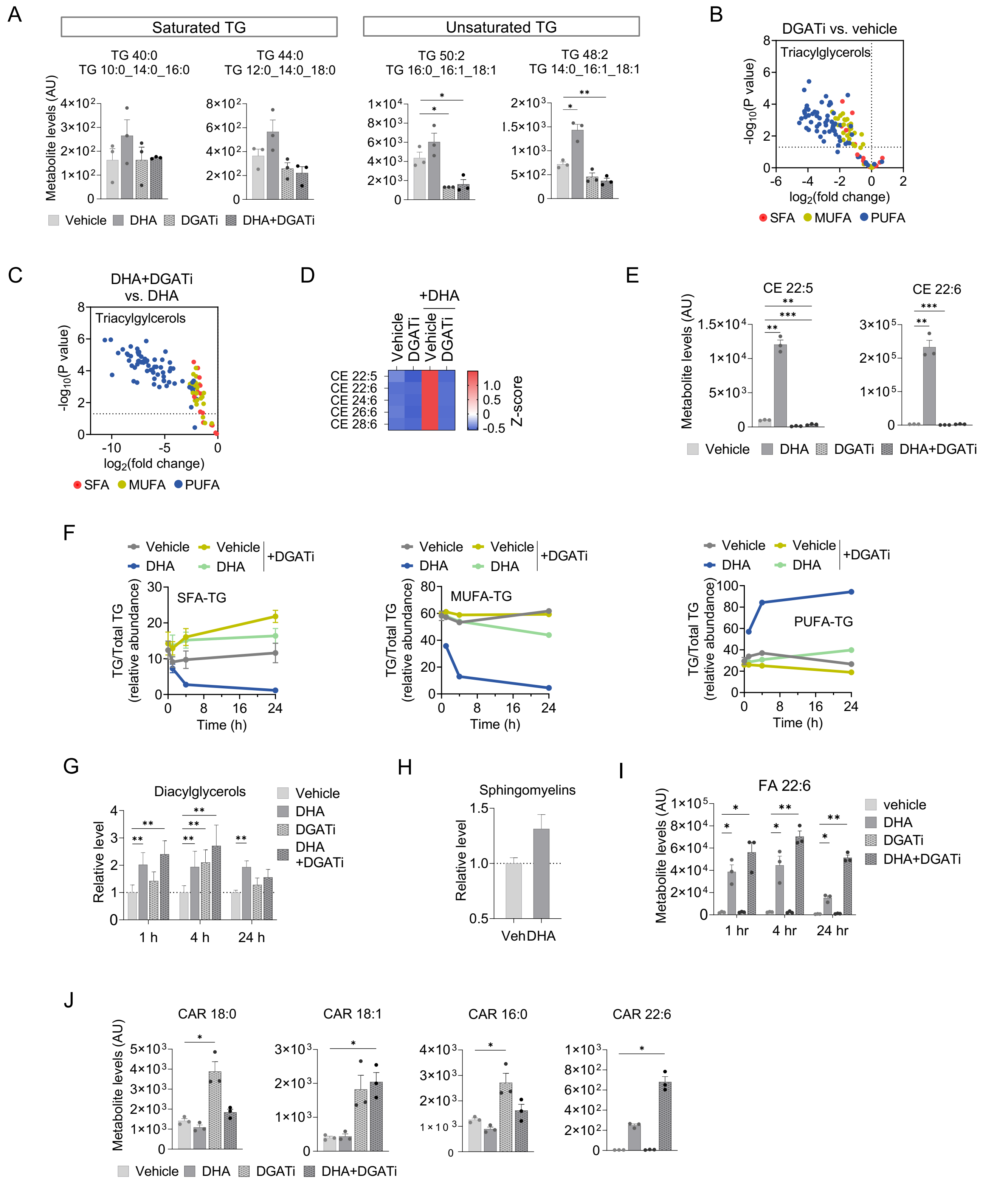

Figure S2. DHA and DGATi-induced changes in LD-stored neutral lipids.

(A-J) MDA-MB-231 cells were supplemented with 25 µM docosahexaenoic acid (DHA) for 1, 4 and 24 h with or without 20 µM DGAT1 and 20 µM DGAT2 inhibitors (DGATi). At each timepoint, cellular lipids were extracted and lipidomics analysis performed.

(A) Abundance of selected saturated and unsaturated TG lipids after 24 h of treatment. Each point represents a biological replicate.

(B, C) Volcano plots of TG species altered by 24 h treatment: (B) with DGATi alone, and (C) with DGATi in DHA-supplemented cells. Lipids are categorized and coloured by unsaturation as indicated in the legend.

(D) Heatmap showing relative abundance of all quantified CEs (n = 5) after 24 h of treatment. Data were Z-score normalized.

(E) Selected CE lipids after 24 h of treatment. Each point represents a biological replicate.

(F) Relative abundance of SFA-, MUFA-, or PUFA-containing TGs.

(G) Relative total levels of diacylglycerols.

(H) Relative total levels of sphingomyelins after 24 h of treatment.

(I) Abundance of free DHA (FA 22:6). Each point represents a biological replicate.

(J) Abundance of selected acylcarnitine (CAR) species after 24 h of treatment.

Data are presented as mean ± SEM (A, E–J) of three independent biological replicates. Significance was determined using unpaired *t*-test. $p > 0.05$ = ns; *$p < 0.05$; **$p < 0.01$; ***$p < 0.001$. Data for volcano plots (B, C) were generated from three independent biological replicates using $\log_2$-transformed fold-change values and multiple unpaired *t*-tests ($p < 0.05$).

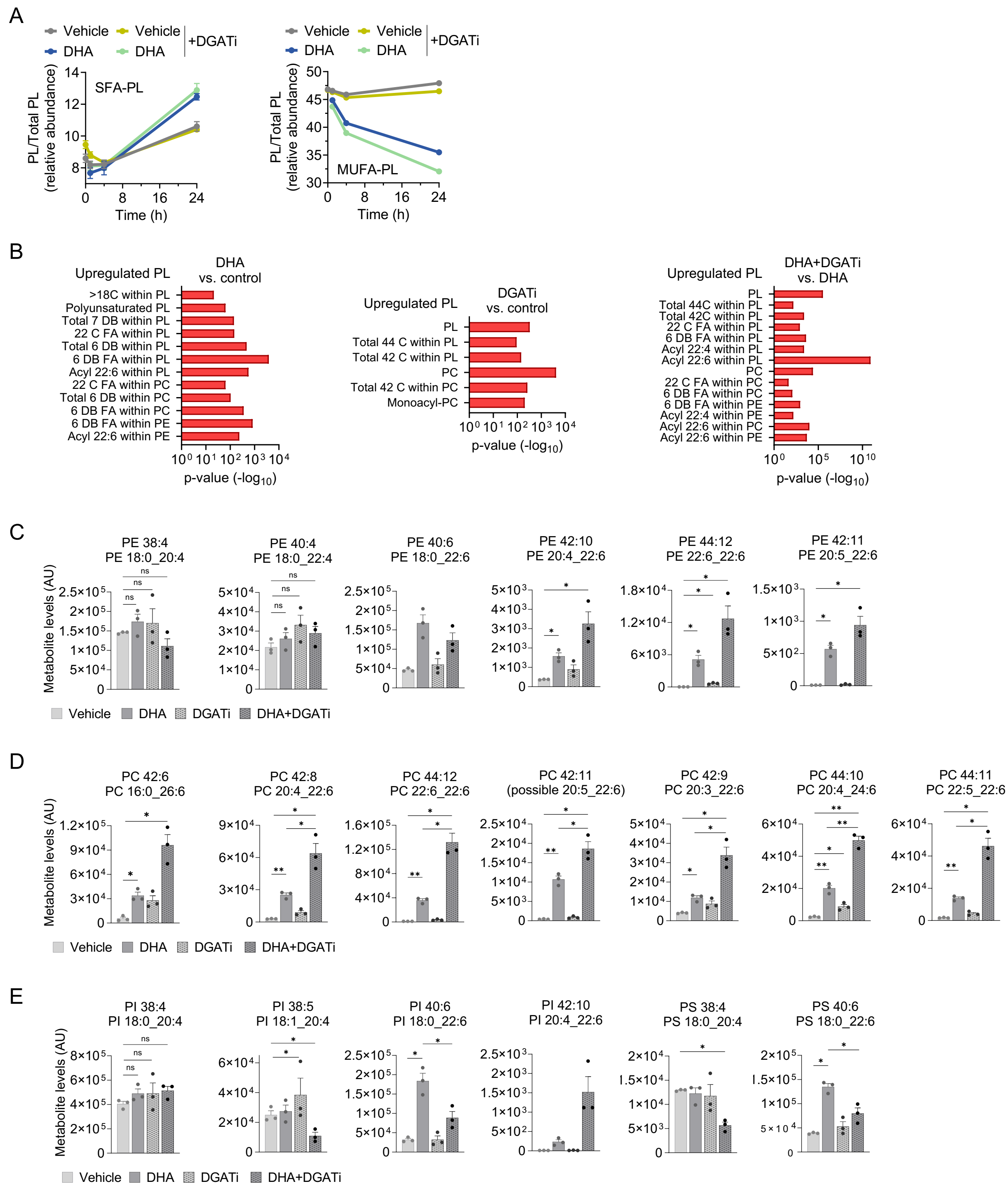

Figure S3. DHA and DGATi-induced changes in PL acyl-chain composition.

(A) Relative abundance of SFA- and MUFA-containing PLs in MDA-MB-231 cells following 1, 4 and 24 h treatment with 25 µM docosahexaenoic acid (DHA) with or without 20 µM DGAT1 and 20 µM DGAT2 inhibitors (DGATi).

(B) Lipid Over-Representation Analysis (LORA) of upregulated PL clusters in MDA-MB-231 cells following the 24 h treatment described in (A). Data for LORA were generated from three independent biological replicates using log_2-transformed fold-change values and multiple unpaired *t*-tests ($p < 0.1$)

(C–E) Abundance of selected PUFA-containing phosphatidylethanolamine (PE) (C), phosphatidylcholine (PC) (D), phosphatidylinositol (PI) and phosphatidylserine (PS) PL species in MDA-MB-231 cells following the 24 h treatment described in (A). Each point represents a biological replicate.

Data are presented as mean ± SEM of three independent biological replicates (A, C–E). Significance was calculated by unpaired *t*-test. $p > 0.05$ = ns; $^{*}p < 0.05$; $^{**}p < 0.01$.

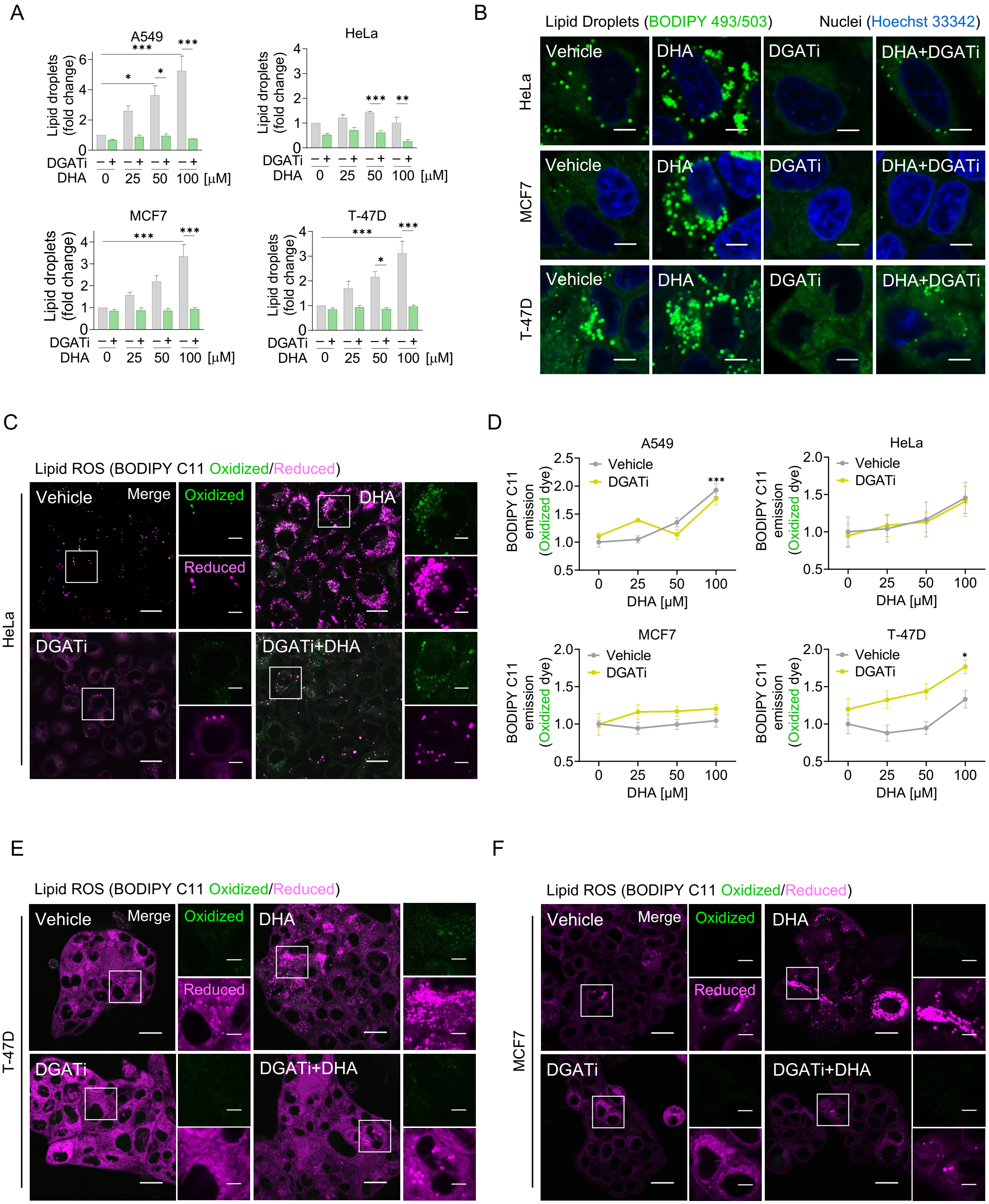

Figure S4. DHA- and DGATi-induced changes in LD and lipid ROS levels in ferroptosis-resistant cells.

(A) Lipid droplet levels measured by Nile Red staining in indicated cell lines treated with 25-100 μM docosahexaenoic acid (DHA) for 24 h, with or without 20 μM DGAT1 and 20 μM DGAT2 inhibitors (DGATi).
(B) Representative confocal microscopy images of HeLa, MCF7 and T-47D cells treated with 100 μM DHA for 24 h, with or without DGATi. Lipid droplets were stained by BODIPY 493/503 and nuclei by Hoechst 33342. Scale bar, 5 μm.
(C) Representative confocal microscopy images of HeLa cells showing oxidized (green) and reduced (magenta) fluorescent signals of the BODIPY C11 581/591 probe induced by the treatment described in (B). Boxed insets show an area approximately covering a single cell in separate fluorescent channels. Scale bars, 20 μm and 5 μm (inset).
(D) Lipid peroxidation assessed by the oxidized BODIPY C11 581/591 probe using flow cytometry in indicated cell lines following the treatment described in (A).
(E, F) Representative confocal microscopy images of T-47D (E) and MCF7 (F) cells showing oxidized (green) and reduced (magenta) fluorescent signals of the BODIPY C11 581/591 probe induced by the treatment described in (B). Boxed insets show an area approximately covering a single cell in separate fluorescent channels. Scale bars, 20 μm and 5 μm (inset).
All data are presented as mean ± SEM of three independent biological replicates (significance was calculated by two-way ANOVA with Tukey adjustment). Microscopic images are representative of two (B, C, E, F) independent biological replicates. $p > 0.05$ = ns; $^{*}p < 0.05$; $^{**}p < 0.01$; $^{***}p < 0.001$.

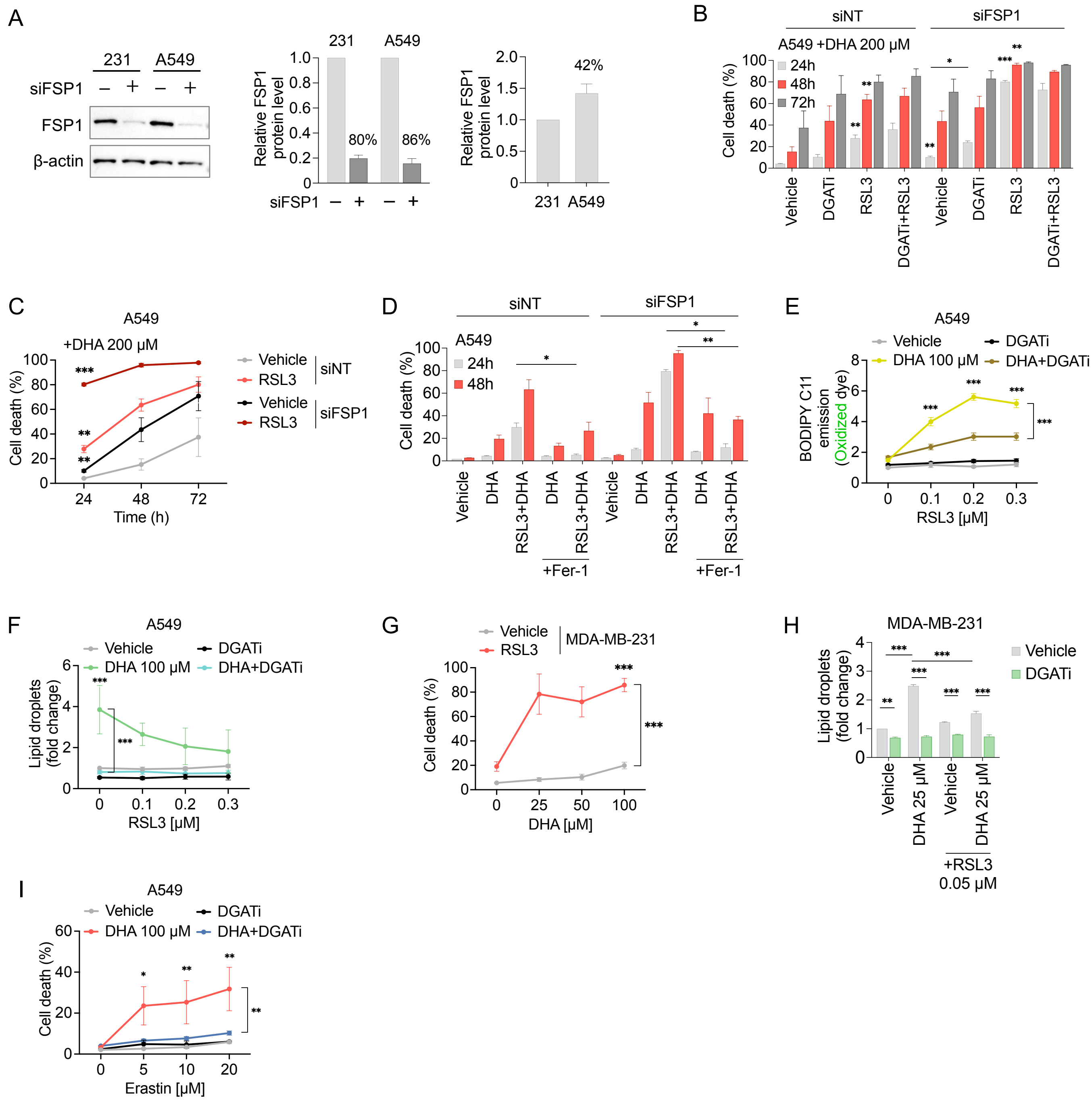

Figure S5. DGAT inhibition protects or promotes ferroptosis depending on the context.

(A) Immunoblot detection of ferroptosis suppressor protein 1 (FSP1) and β-actin in whole-cell lysates of control (siNT) and FSP1-silenced (siFSP1) MDA-MB-231 and A549 cells prepared 48 h after reverse transfection. Representative western blot is shown and densitometric analysis of three biological replicates.

(B) Cell death (%) determined by TMRM/YO-PRO-1 staining in siNT and siFSP1 A549 cells treated with 200 μM docosahexaenoic acid (DHA) for 24, 48 and 72 h, with or without 20 μM DGAT1 and 20 μM DGAT2 inhibitors (DGATi) and 0.1 μM RSL3.

(C) Cell death (%) in siNT and siFSP1 A549 cells treated with 200 μM DHA for 24, 48 and 72 h, with or without 0.1 μM RSL3.

(D) Cell death (%) in siNT and siFSP1 A549 cells treated with 200 μM DHA for 24 and 48 h, with or without 0.1 μM RSL3 and 5 μM ferrostatin-1 (Fer-1).

(E) Lipid peroxidation assessed by BODIPY C11 581/591 using flow cytometry in A549 cells treated with 100 μM DHA for 24 h, with or without DGATi and 0.1, 0.2 or 0.3 μM RSL3.

(F) Lipid droplet levels measured by Nile Red staining in A549 cells following the treatment described in (C).

(G) Cell death (%) in MDA-MB-231 cells treated for 24 h with 25, 50 or 100 μM DHA, with or without 0.05 μM RSL3.

(H) Lipid droplet levels measured by Nile Red staining in MDA-MB-231 cells treated with 25 μM DHA for 24 h, with or without DGATi and 0.05 μM RSL3.

(I) Cell death (%) in A549 cells treated with 100 μM DHA for 24 h, with or without DGATi, and 5, 10 or 20 μM erastin.

All data are presented as mean ± SEM of two (C, D) or three (B, E, F, H, I) or four (G) independent biological replicates. Significance was calculated by unpaired *t*-test (B, C) or two-way ANOVA with Tukey adjustment (D–I). $p > 0.05$ = ns; *$p < 0.05$; **$p < 0.01$; ***$p < 0.001$.

Table S1. Features of cancer cell lines used in this study.

Features	MDA-MB-231	T-47D	MCF7	A549	HeLa
**Tissue Origin & Molecular Subtype**	Breast (Carcinoma), Triple-negative / Basal-like (ER-/PR-/HER2-)	Breast (Carcinoma), Luminal A (ER+/PR+/HER2-)	Breast (Carcinoma), Luminal A (ER+/PR+/HER2-)	Lung (Adenocarcinoma, Non-Small Cell)	Cervix (Adenocarcinoma)
**Key Oncogenic Drivers**	*KRAS*^{G13D}, *TP53, BRAF, CDKN2A*	*PIK3CA, TP53*	*PIK3CA, ESR1, GATA3*	*KRAS*^{G12S}, *KEAP1*	*MYC, TP53, RB1*
**Lipid Metabolic Profile**	↑FA uptake [71,72], ↑Mitochondrial FAO [73–75], ↑Membrane unsaturation [36,37,64,75,76]	↑DNL via PIK3CA activation [77], ↓Membrane unsaturation [36,64,75,76]	↑DNL via PIK3CA activation [77], ↓Membrane unsaturation [36,64,75,76]	↑DNL via KRAS activation [78–80], ↑OXPHOS [81], ↓Membrane unsaturation [80,82,83]	↑Lipolysis [54]
**Membrane Remodelling Regulators**	↑ACSL4 [64,76], ↓ACSL3 [75], ↓MBOAT1 [36], ↑FADS1/2 [37]	↓ACSL4 [64,76], ↑ACSL3 [75], ↑MBOAT1 [36]	↓ACSL4 [64,76], ↑ACSL3 [75], ↑MBOAT1 [36]	↓ACSL4 [83], ↑ACSL3 [82,83], ↑FASN [78,80]	Not extensively characterized
**Redox Profile**	↓GPX4 [29], ↑FSP1 [29], ↓Antioxidant capacity (no direct Nrf2 activation) [84–86]	↑GPX4 [29], ↓FSP1 [29], ↑Antioxidant capacity (via PIK3CA-driven Nrf2 upregulation) [84,87–89]	↑GPX4 [29], ↓FSP1 [29], ↑Antioxidant capacity (via PIK3CA-driven Nrf2 upregulation) [84,87–89]	↓GPX4 [29,90], ↑FSP1 [29,90–92], ↑Antioxidant capacity (Nrf2 hyperactivation due to KEAP1 loss) [93,94]	↓GPX4 [95], ↓FSP1 [95], ↓Antioxidant capacity (no direct Nrf2 activation)
**Ferroptosis Sensitivity**	Sensitive	Resistant	Resistant	Resistant	Resistant
**PUFA Tolerance**	Low	High	High	High	High
**Culture Medium**	RPMI-1640 + 10% FBS	RPMI-1640 + 0.2 U/ml human insulin + 10% FBS	EMEM + 0.01 mg/ml human insulin + 10% FBS	DMEM/F-12 + 10% FBS	DMEM/GlutaMAX+ 10% FBS

Abbreviations: ACSL = acyl-CoA synthetase, long-chain, DNL = de novo lipogenesis, ER = estrogen receptor, FADS = fatty acid desaturase, FAO = fatty acid oxidation, FASN = fatty acid synthase, FBS = fetal bovine serum, HER2 = human epidermal growth factor receptor 2, KEAP1 = Kelch-like ECH-associated protein 1, MBOAT = membrane bound O-acyl transferase, Nrf2 = nuclear factor erythroid 2-related factor 2, OXPHOS = oxidative phosphorylation, PR = progesterone receptor, TNBC = triple-negative breast cancer.

Table S2. List of siRNA sequences used in this study.

Target	Product name	Catalog no.	siRNA sequence
**N/A**	AllStars Negative Control siRNA	1027281	Proprietary to Qiagen
**AIFM2 (FSP1)**	Hs_AMID_1	SI00144445	CTGCACCGGCATCAAGATCAA
	Hs_AMID_4	SI00144466	CTCGGTGACTTTCAAGGACAA
	Hs_AMID_5	SI03069276	CAGGACCTTCGGCCTAAGAAA
	Hs_AMID_6	SI03082541	CCGGGCAAGTTTAATGAGGTT
